# Supplementary material for: Inkjet-printed unclonable quantum dot fluorescent anti-counterfeiting labels with artificial intelligence authentication
Source: Nat Commun. 2019 Jun 3;10:2409. doi: 10.1038/s41467-019-10406-7 (PMC6547729; doi:10.1038/s41467-019-10406-7)
Supplement: Supplementary file 1 — Supplementary Information [file 41467_2019_10406_MOESM1_ESM.pdf]

Supplementary Information

Inkjet-printed unclonable quantum dot fluorescent anti-counterfeiting labels with artificial intelligence authentication

Liu et al.

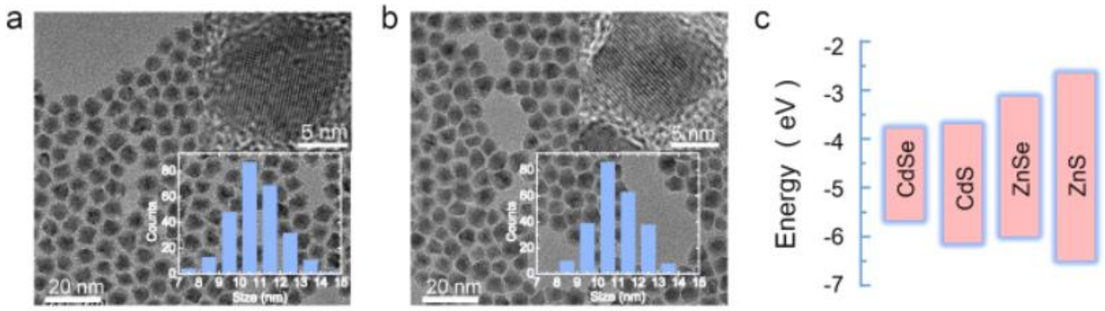

Supplementary Figure 1. Typical TEM/HRTEM images and corresponding particle size distributions of (a) green and (b) blue core-shell quantum dots. (c) Energy levels of CdS, CdSe, ZnSe and ZnS. The valence and conduction bands values are taken from literature. [1]

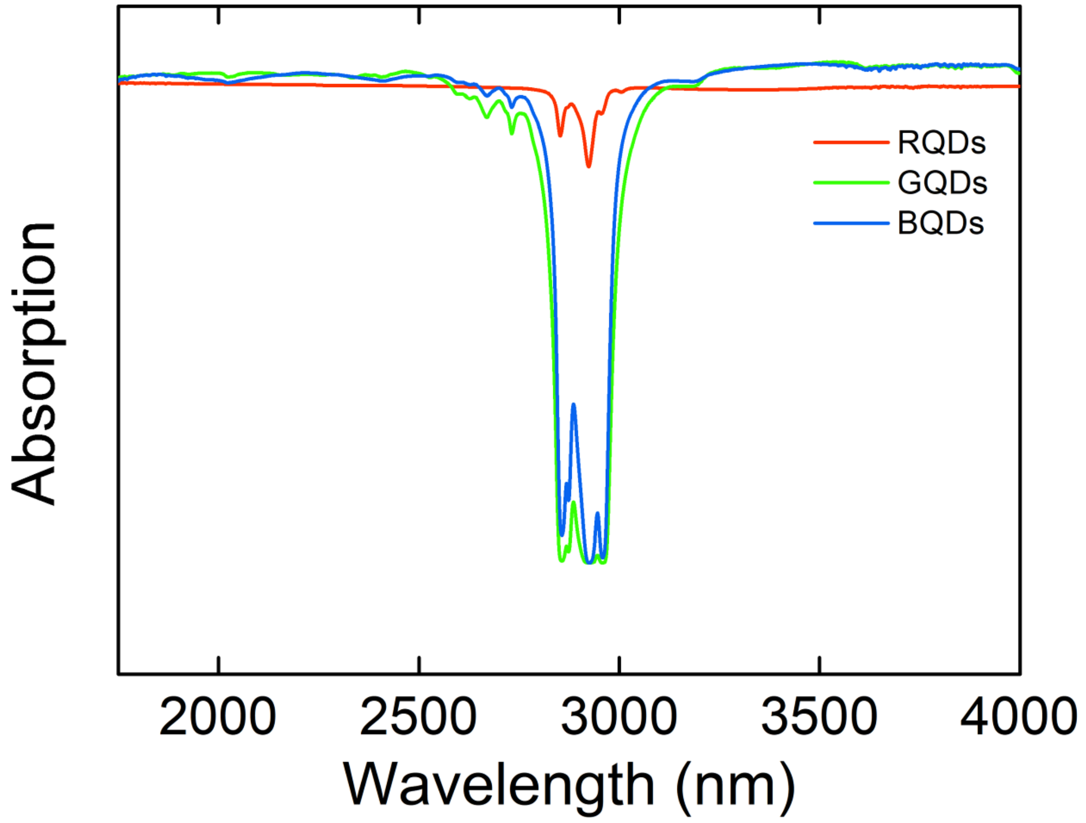

Supplementary Figure 2. Fourier transform infrared (FTIR) spectra of the red, green and blue emission quantum dots (QDs).

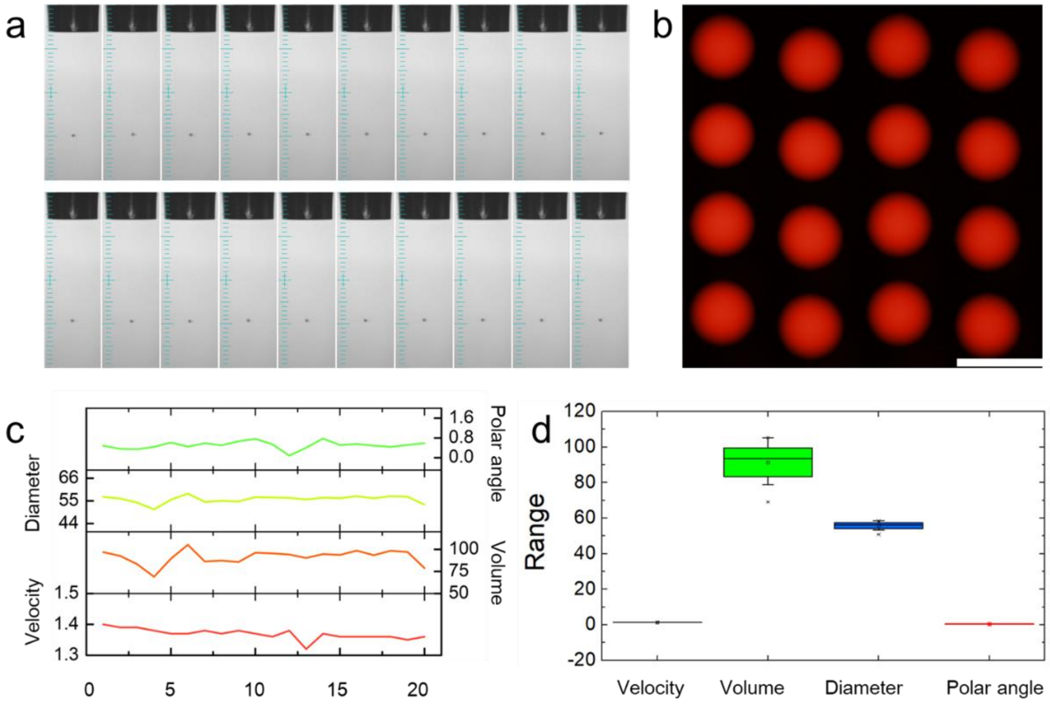

Supplementary Figure 3. (a) Photographs of 20 stable single droplets (red quantum dot inks) obtained at the capture time of 200 μs. The printing process between any two adjacent points was stop more than 30 s to test the stability of the inks. (b) Fluorescence photographs of the inkjet-printed droplets on PMMA layer at the first stage (scale bar: 200 um). (c, d) the velocity, volume, diameter and polar angle curves and error bars of the 20 droplets. Error bar, standard deviation of corresponding parameters. The results indicate that the unrepeatable patterns formed are not originated from inkjet printing technique or the droplets hit to substrate at the initial time.

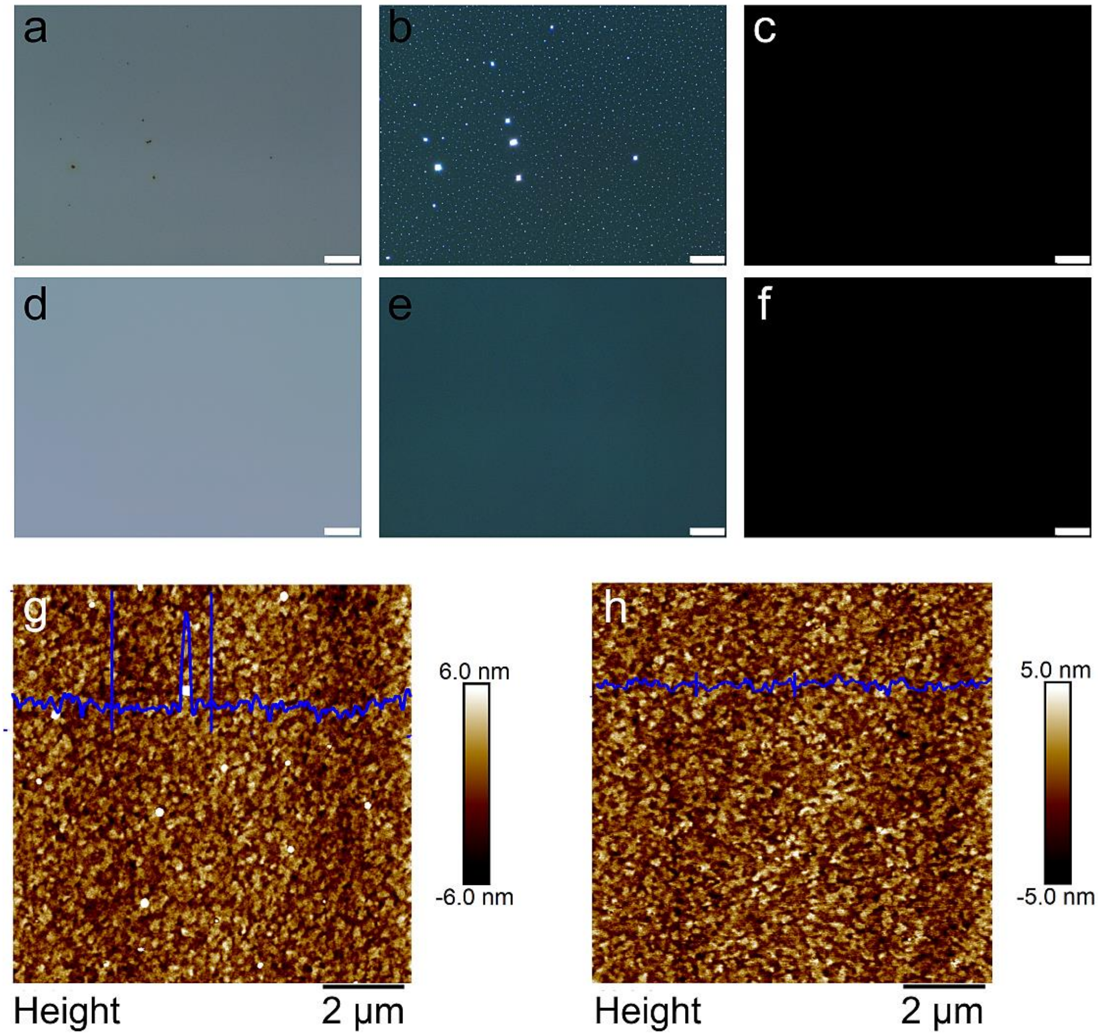

Supplementary Figure 4. (a, d) Bright field, (b, e) dark-field scattering, (c, f) fluorescence microscopy images at the irradiation of UV light (scale bar: 20 um) and (g, h) atom force microscope (AFM) images of spin-coated rough and smooth PMMA surface modification. The obvious white points accord to the pinning points in inkjet printing.

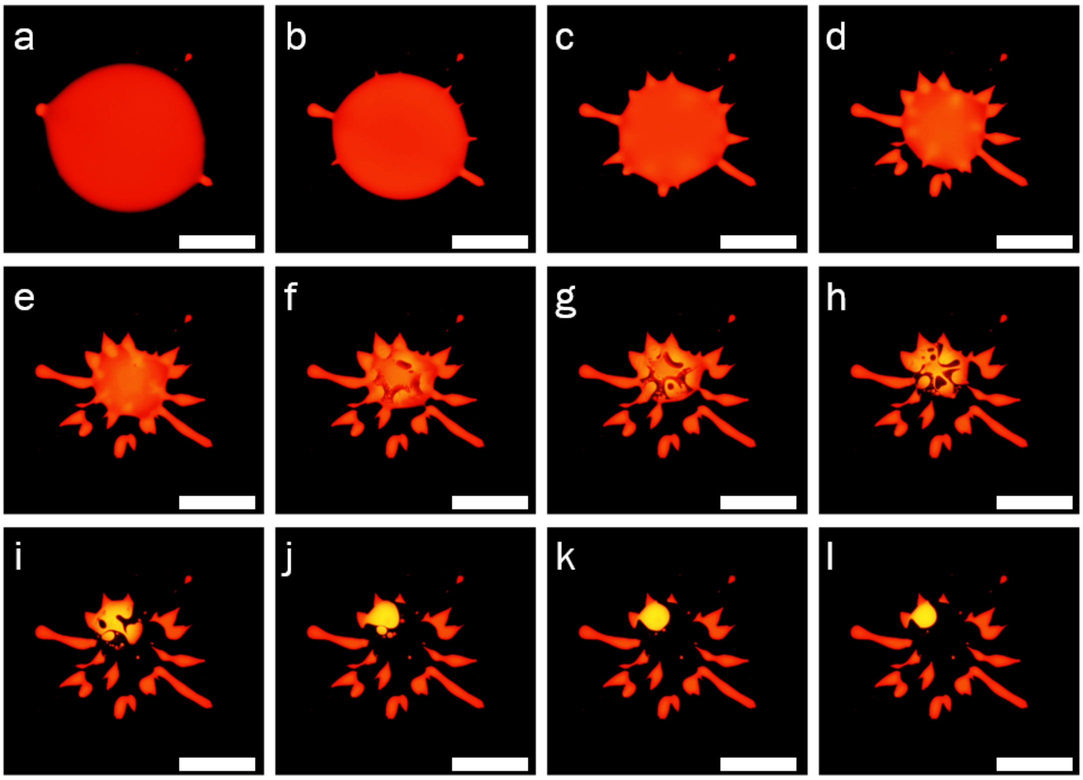

Supplementary Figure 5. (a-l) In-situ fluorescence microscopic images of the red inks droplets moving process in the evaporation period from t_0+0 s to t_0+300 s. The scale bar is 30 μm.

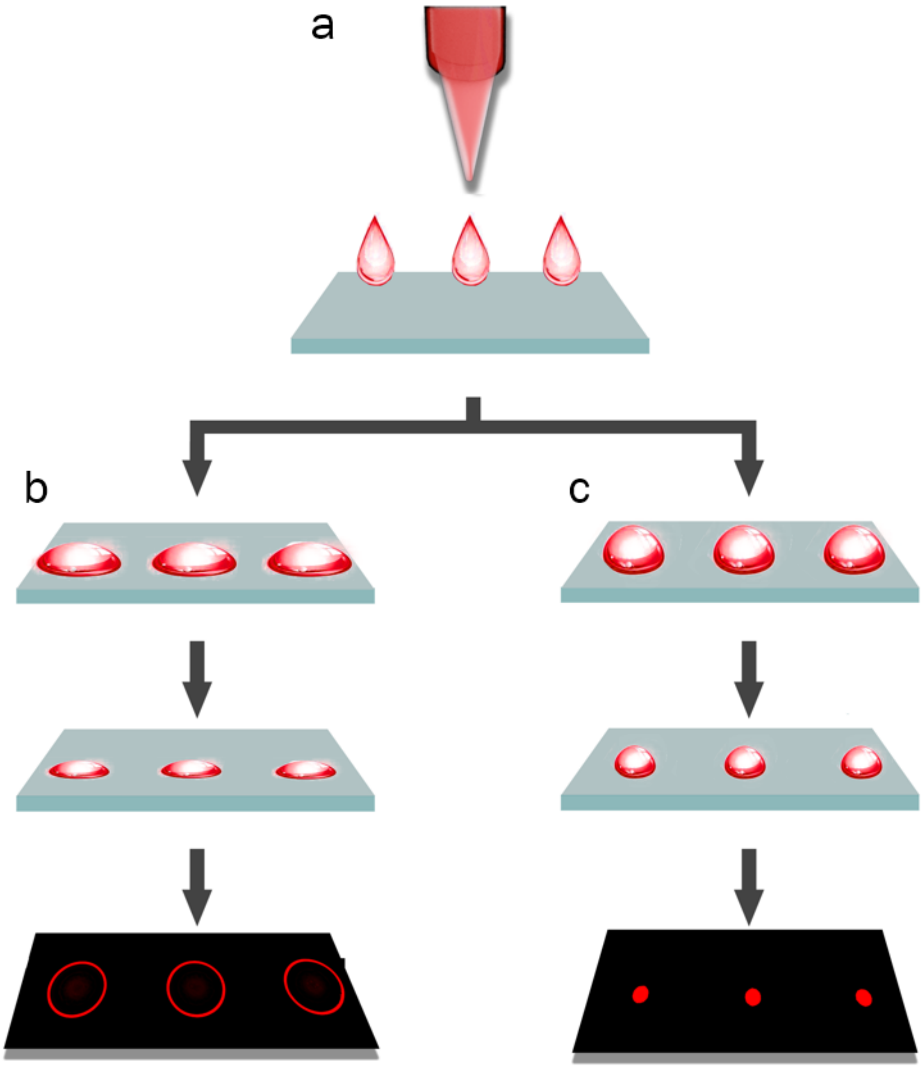

Supplementary Figure 6. Schematics illustrating (a) inkjet printing and evaporation processes at (b) without PMMA-modified hydrophilic surface and (c) smooth PMMA-modified hydrophilic surface.

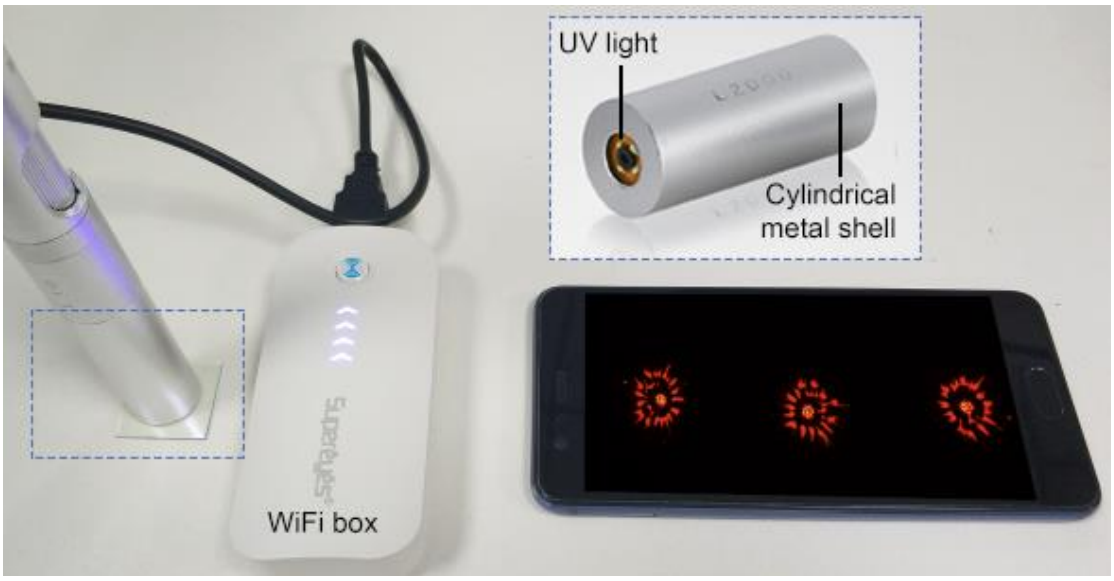

Supplementary Figure 7. Pattern readout with a smartphone microscope: the portable mini-microscope is composed of a UV chip, a 200× magnification-adjustable objective lens covered with a cylindrical metal shell and a small WiFi box. The insert is an enlarged view of the light source and the lens.

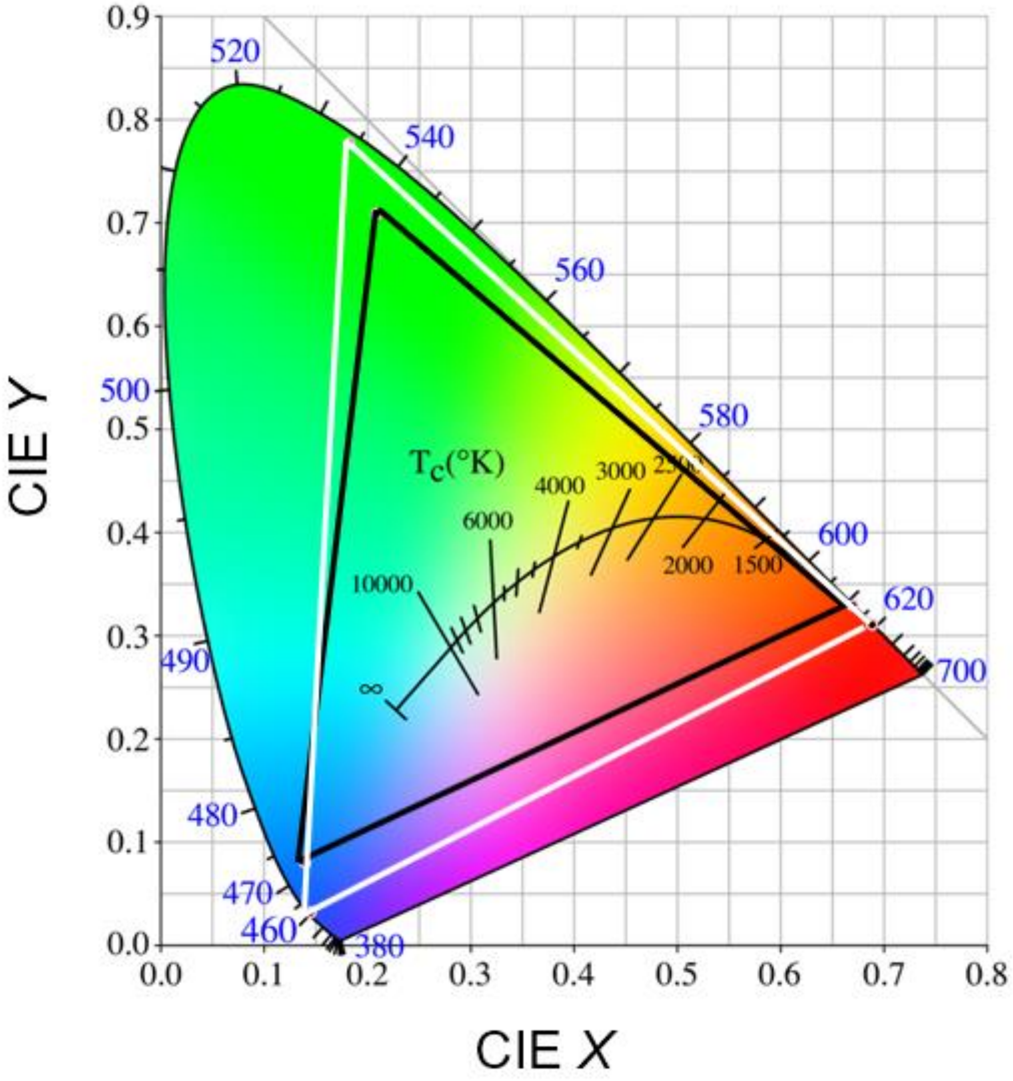

Supplementary Figure 8. Commission internationale de l'éclairage (CIE) 1931 color coordinate triangle of blue, green, and red monochromatic light.

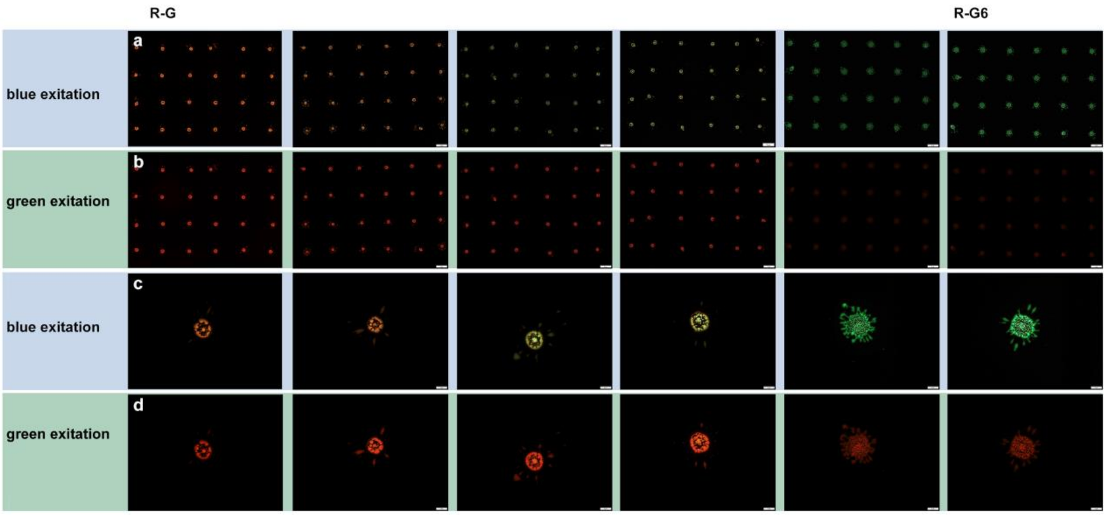

Supplementary Figure 9. Fluorescence microscope photographs of gradient color shift from red to green, corresponding to the increasing ink component ratios (the mixture of red and green quantum dots) of green quantum dots from left to right. (a) and (b) correspond to fluorescence microscope photographs under blue and green light excitation with the scale bars of 200 μm. (c) and (d) (scale bars: 50 μm) is enlarged image corresponding to the points in row 2, column 3 in (a) and (b).

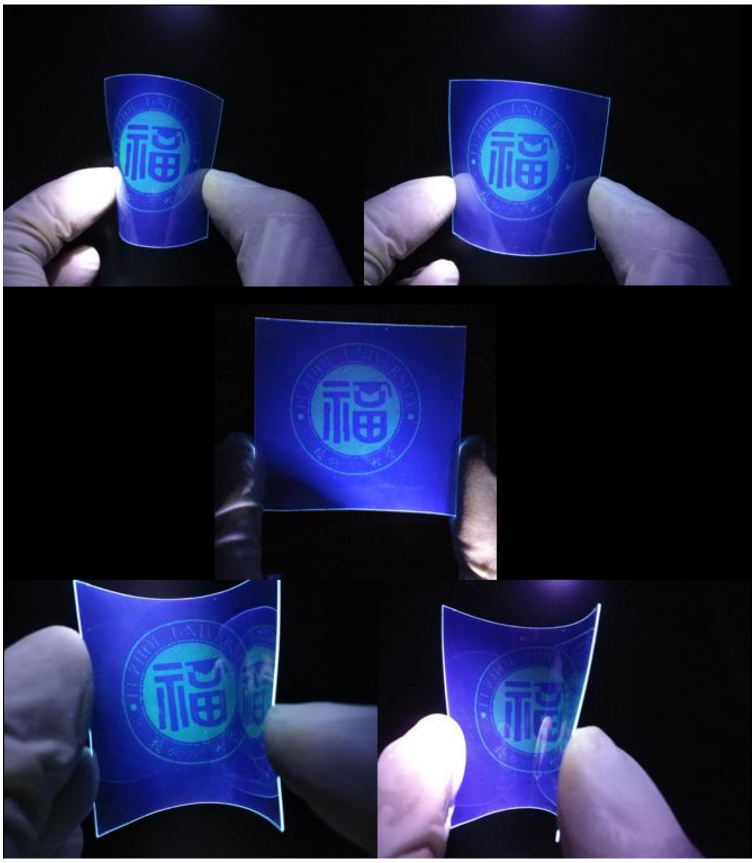

Supplementary Figure 10. Fluorescence images of inkjet-printed pattern on flexible substrate.

The Fuzhou University logo is adapted with permission of the Fuzhou University.

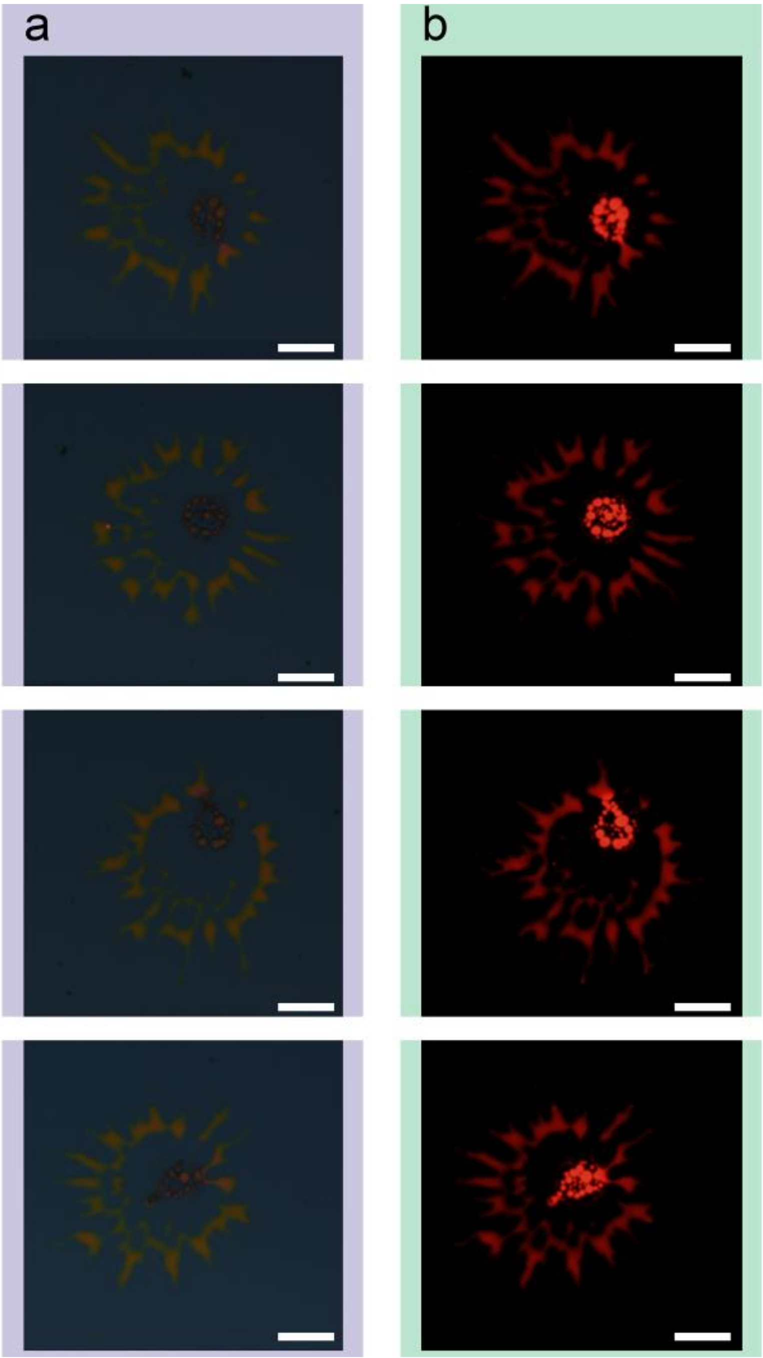

Supplementary Figure 11. Microscopic images at the irradiation of (a) white and (b) blue light corresponding to Figure 3g (scale bar: 20 μm).

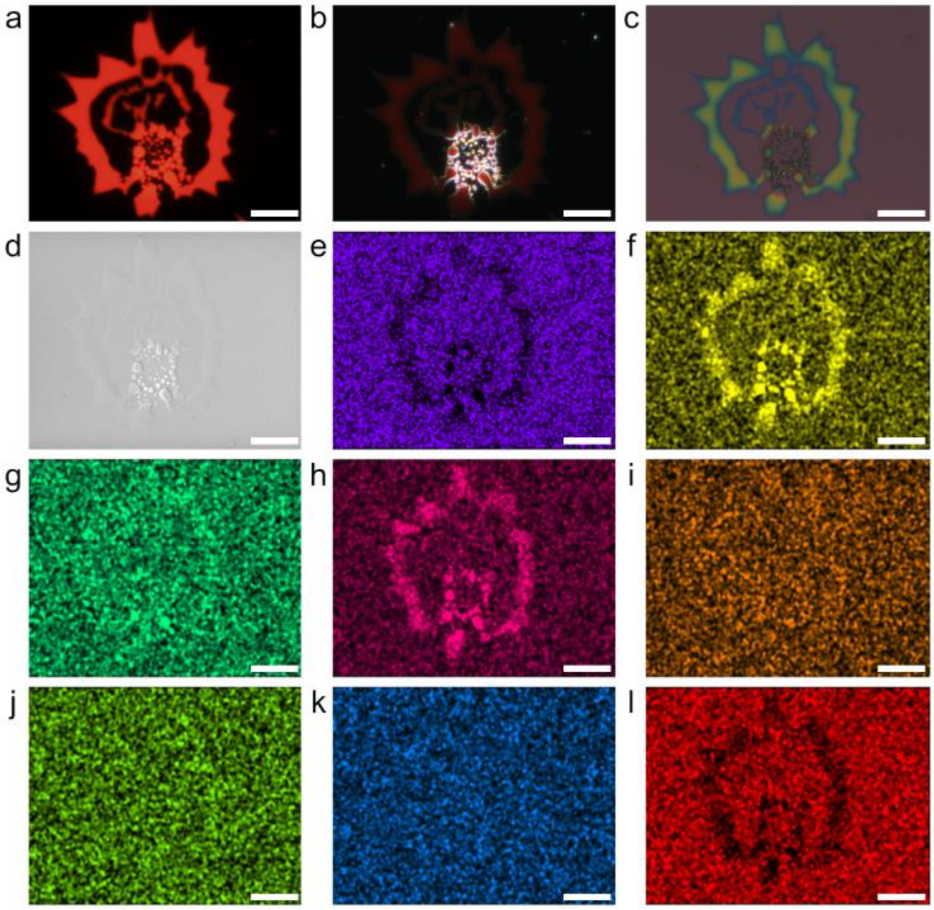

Supplementary Figure 12. Microscope images and energy dispersive X-ray spectroscopy mapping of red quantum-dot point. (a) Fluorescence, (b) dark-field and (c) bright-field microscope images of red point by inkjet printing on designed PMMA layer. The corresponding (d) SEM image, and corresponding maps of (e) Si, (f) Cd, (g) Se, (h) S, (i) Zn, (j) Au, (k) W and (l) O (scale bar: 20 um). The microscope images are consistent with energy dispersive X-ray spectroscopy mapping of red quantum dots very well, indicating the dependability and scientific basis of the characterization analysis using microscope images. Si and O are chosen as the dominant components in substrate, W and Au are elected to eliminate the unavoidable based noise of the SEM instrument.

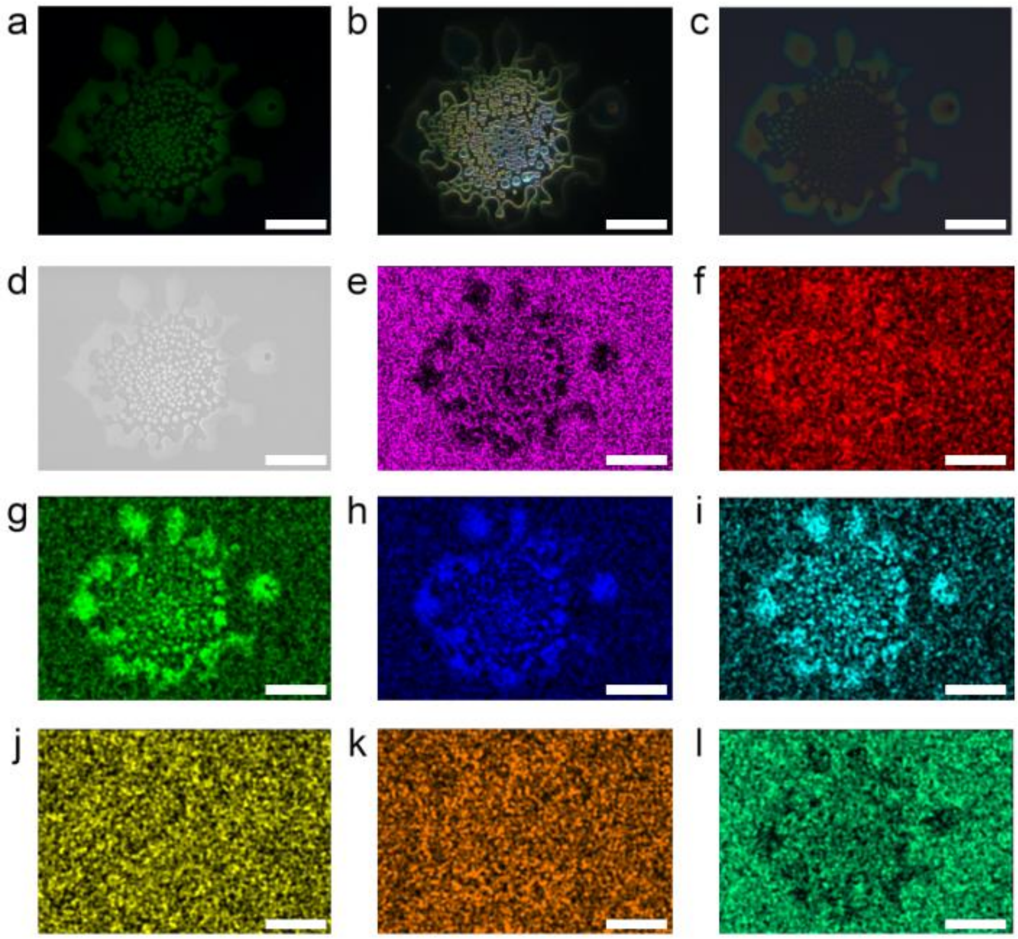

Supplementary Figure 13. Microscope images and energy dispersive X-ray spectroscopy mapping of green quantum-dot point. (a) Fluorescence, (b) dark-field and (c) bright-field microscope images of red point by inkjet printing on designed PMMA layer. The corresponding (d) SEM image, and corresponding maps of (e) Si, (f) Cd, (g) Se, (h) S, (i) Zn, (j) Au, (k) W and (l) O (scale bar: 20 um). The microscope images are consistent with energy dispersive X-ray spectroscopy mapping of green quantum dot very well, indicating the dependability and scientific basis of the characterization analysis using microscope images. Si and O are chosen as the dominant components in substrate, W and Au are elected to eliminate the unavoidable based noise of the SEM instrument.

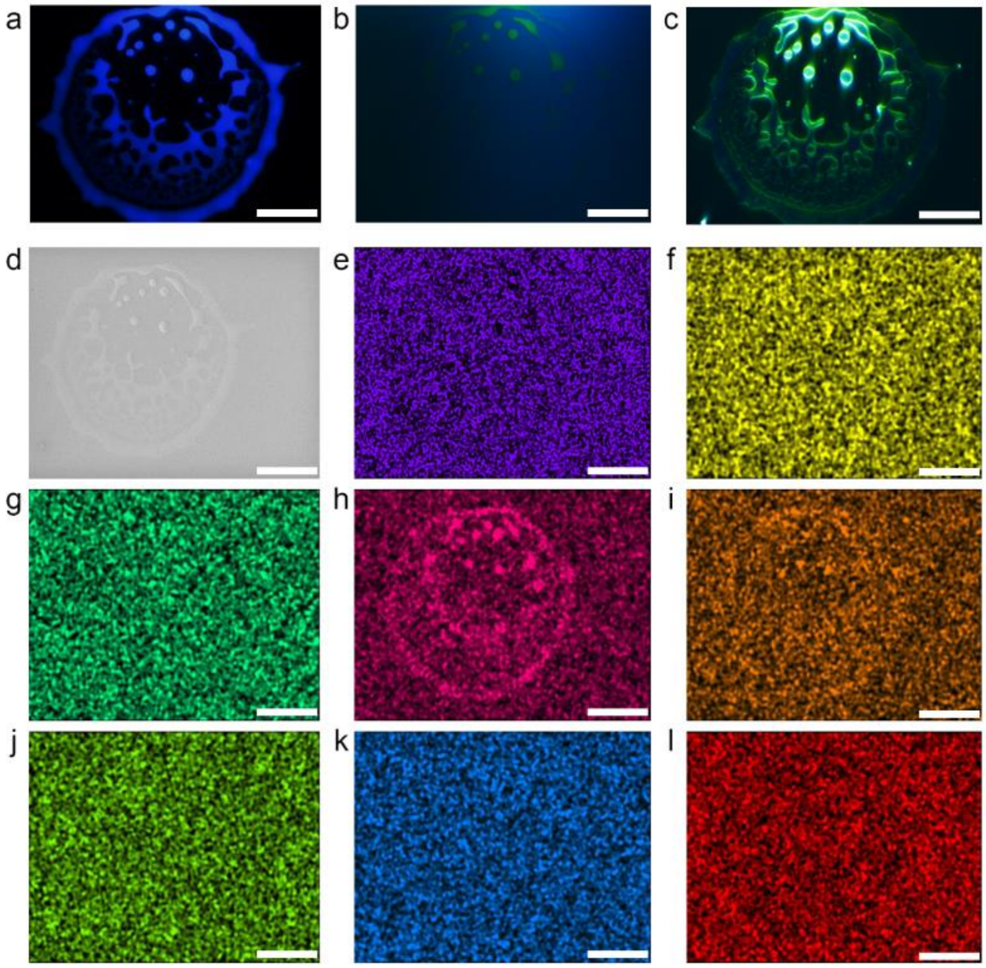

Supplementary Figure 14. Microscope images and energy dispersive X-ray spectroscopy mapping of blue quantum-dot point. (a) Fluorescence, (b) dark-field and (c) bright-field microscope images of red point by inkjet printing on designed PMMA layer. The corresponding (d) SEM image, and corresponding maps of (e) Si, (f) Cd, (g) Se, (h) S, (i) Zn, (j) Au, (k) W and (l) O (scale bar: 20 um). The microscope images are consistent with energy dispersive X-ray spectroscopy mapping of blue quantum dots very well, indicating the dependability and scientific basis of the characterization analysis using microscope images. Si and O are chosen as the dominant components in substrate, W and Au are elected to eliminate the unavoidable based noise of the SEM instrument.

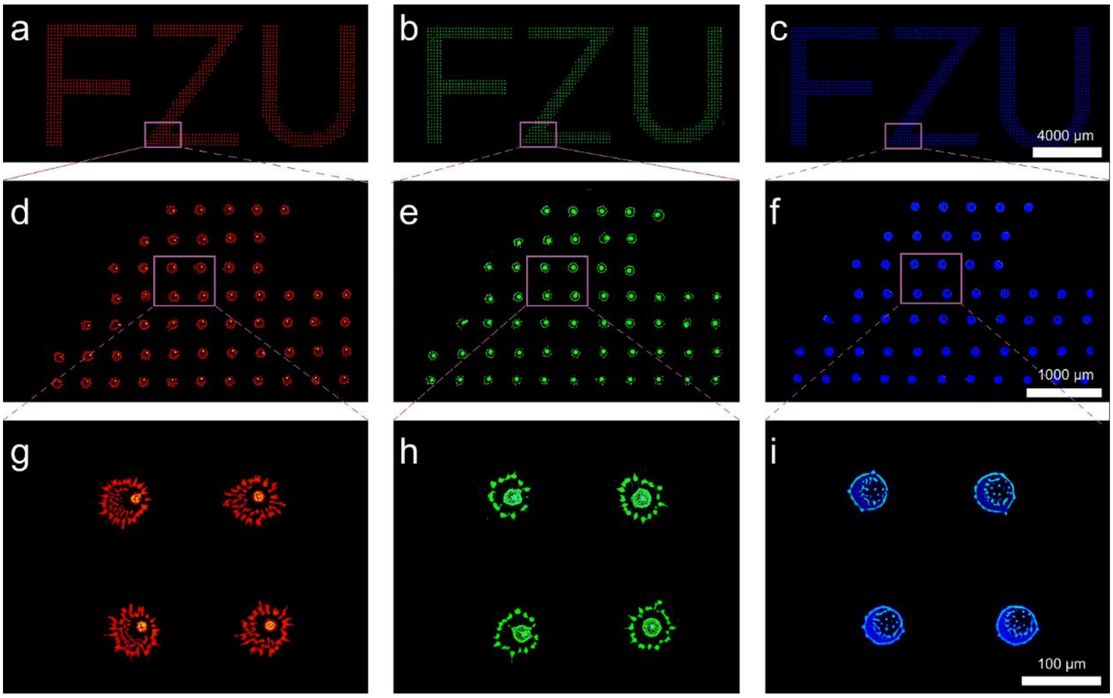

Supplementary Figure 15. Copied photographs of two parallel inkjet-printed patterns at different amplification corresponding to Figure 3. (a-c) high-resolution FZU text pattern. (d-f) enlarged images of yellow box in a-c. (g-i) corresponding enlarged images of yellow box in d-f. The “FZU” text patterns (for red, green and blue quantum dots respectively) inkjet-printed in seemingly identical condition are identical on macro-scale but distinct at the micro-scale; the points on the same sample are also unique.

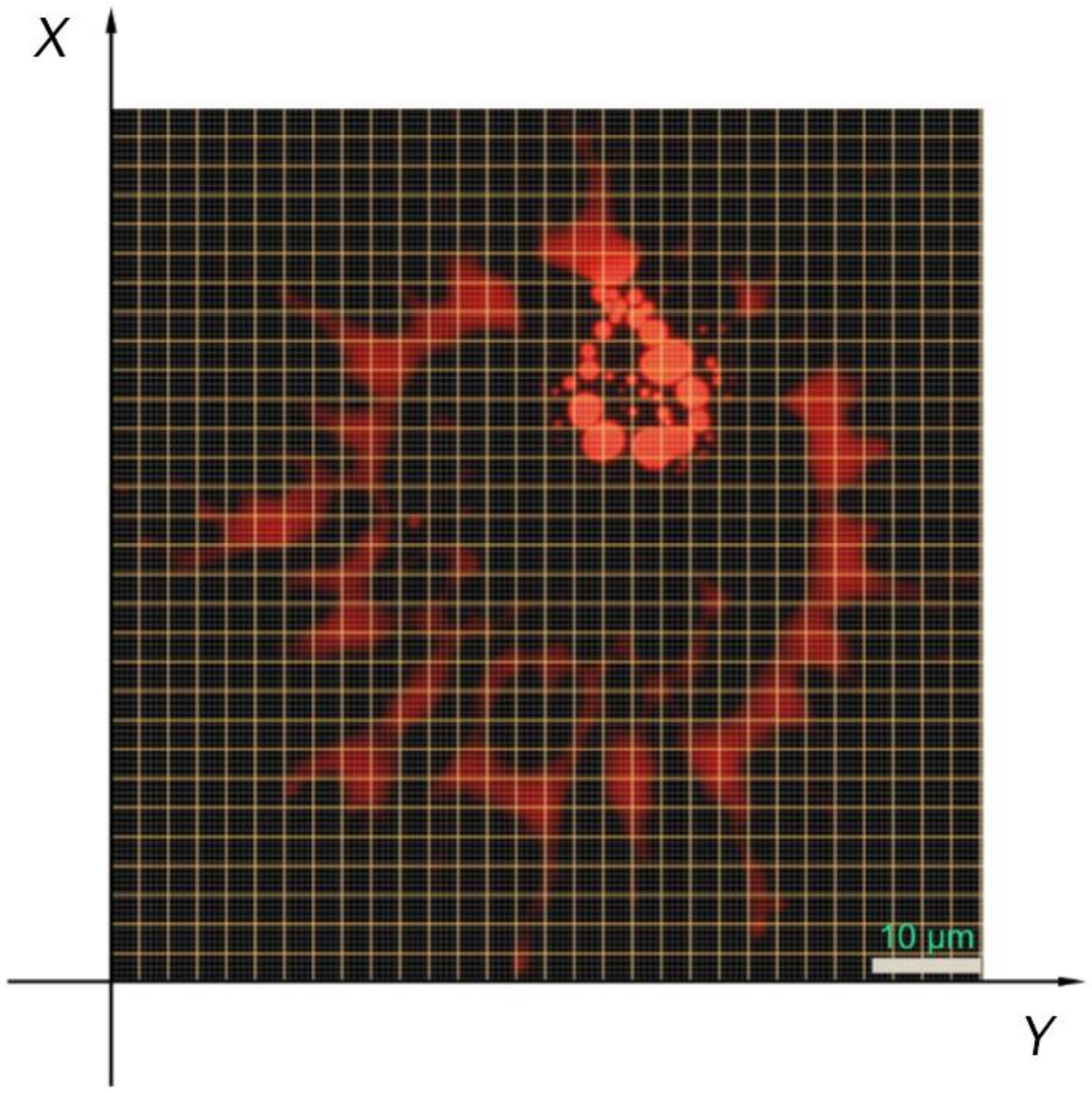

Supplementary Figure 16. An example of the encoding capacity estimation of a red flower-like pattern: R = 150, L = 5, and D = 9/25. Calculation of the encoding capacity is provided detailly Supplementary in Note 2.

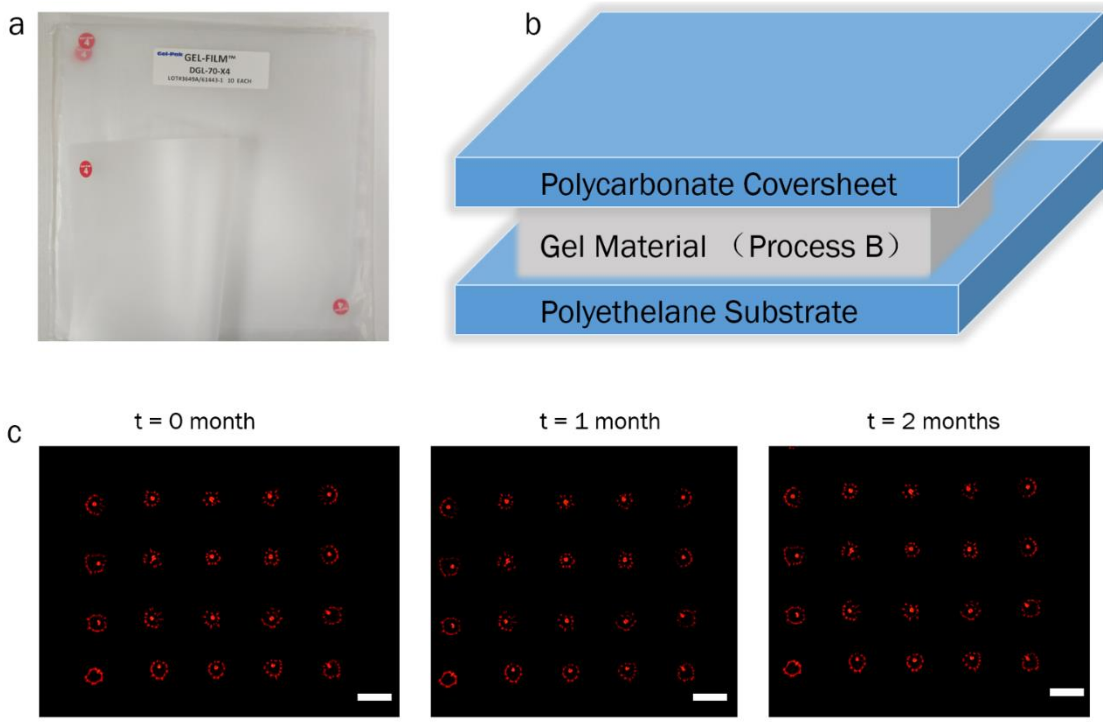

Supplementary Figure 17. Photographs of (a) optically transparent gel films with gel thickness of 1.5 mm and retention level of X4 (purchased from Gel-Pak) and (b) schematic of its structure. (c) Fluorescence images of the security label protected by the sticky gel film. The sample is stable at long-range time (scale bar: 100 μm).

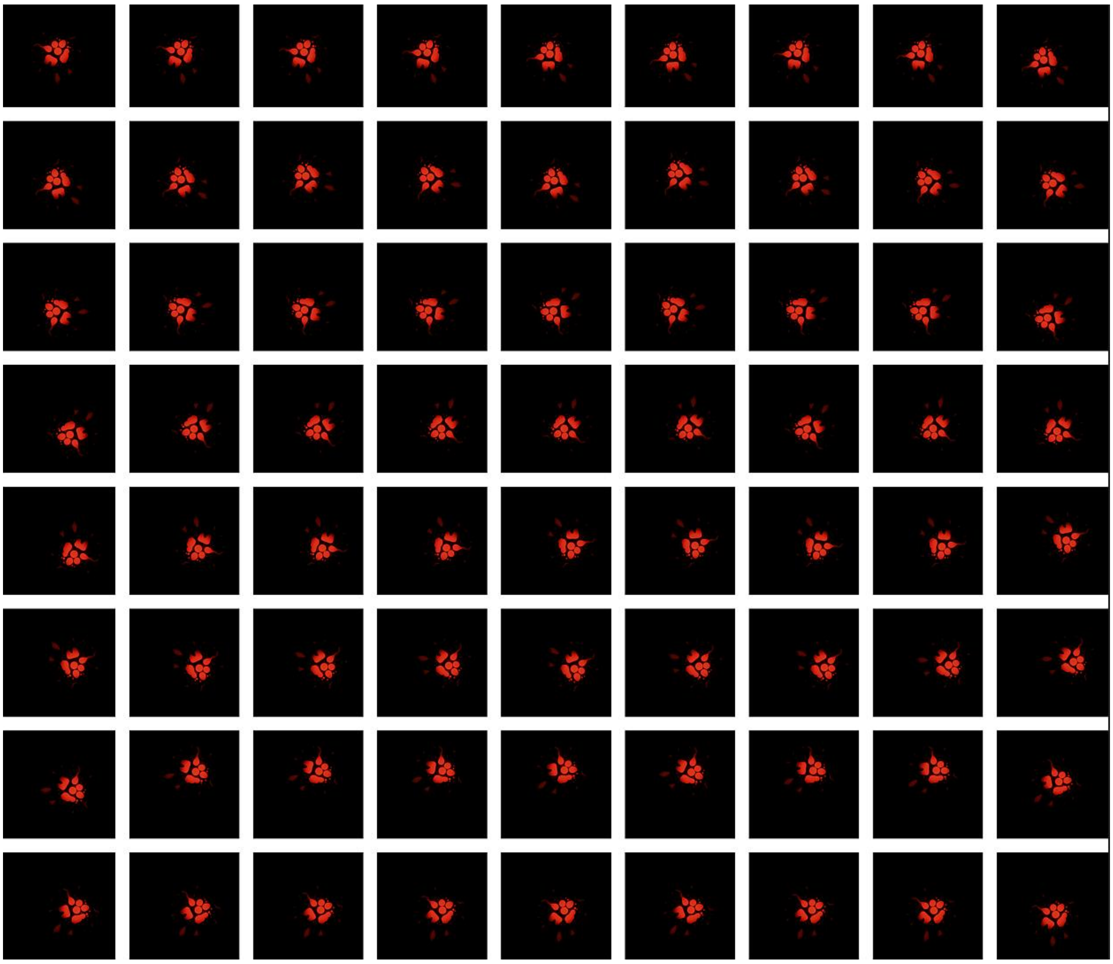

Supplementary Figure 18. Selected 72 images (corresponding to g1) captured for deep learning.

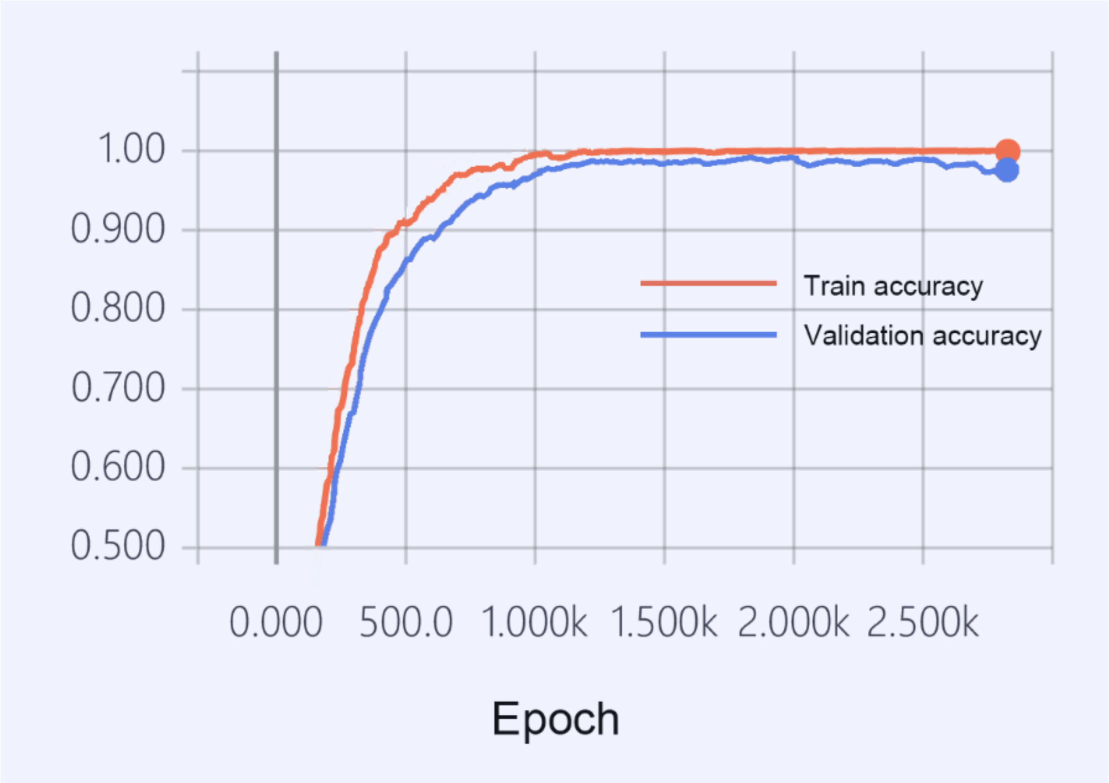

Supplementary Figure 19. Deep learning plots of train and validation accuracy on the training datasets overtraining epochs for authenticating security labels.

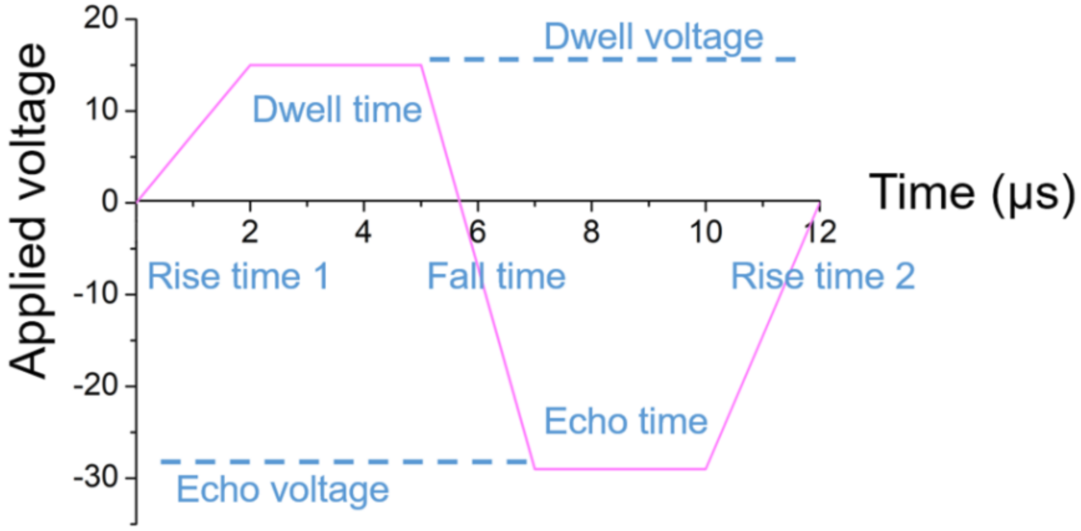

Supplementary Figure 20. Driving voltage waveforms to the inkjet printing nozzle.

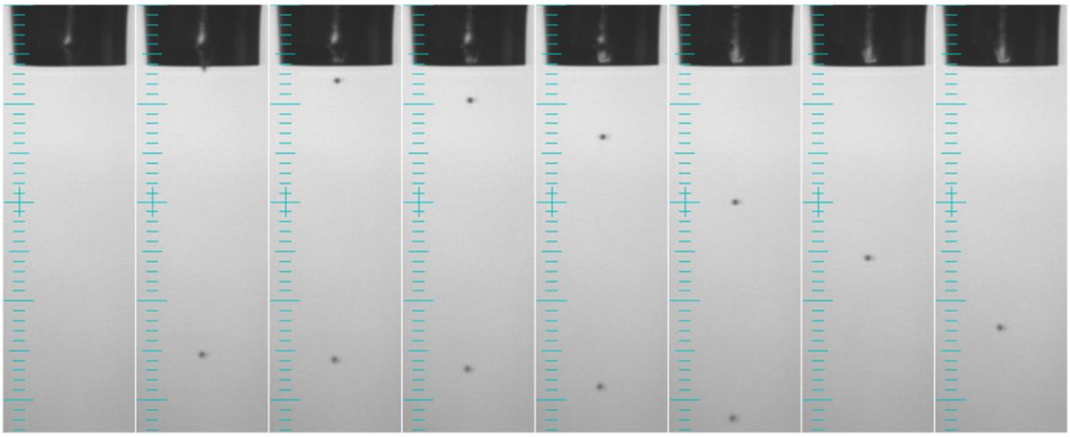

Supplementary Figure 21. Photographs of non-satellite-point and stable single droplet (red quantum-dot inks) obtained at different capture time.

Supplementary Table 1. Statistical results of the relationship between the threshold values and the rate of false positives for AI authentication.

Threshold values	0.4	0.5	0.6	0.7	0.8	0.9
Rate of false positives	2%	0%	0%	0%	0%	0%

Supplementary Table 2. Comparison of our pattern readout tools and authentication methods with those of the previously reported works

No.	Unclonable property	*Readout tools/light source*	Authentication method	Ref
1	Yes	Research-based microscope/ 532 nm laser	Conventional	2
2	Yes	Research-based microscope/ 465, 488, and 450 nm laser	Conventional	3
3	Yes	**Industrial camera/ambient light**	Conventional	4
4	Yes	Research-based microscope/ Hg–Xe lamp	Conventional	5
5	Yes	Research-based microscope/ halogen lamp	Conventional	6
6	Yes	TFT current test machine	Conventional	7
7	**No**	**Naked eye**	Conventional	8
8	Yes	Research-based microscope/ 785 nm laser	Conventional	9
9	Yes	Research-based microscope	Conventional	10
9	Yes	Portable mini-microscope/LED	**AI**	**This work**

Supplementary Notes 1. Calculation of the encoding capacity

To simplify the encoding capacity calculation, we assume that AI defines the xy axis as shown in Supplementary Figure 16. We divide the pattern into 30×30 arrays, in which a unit is further divided into 5×5 arrays of subunits (i.e., Length of each unit, L = 5; Resolution, R = 150). As long as a color (e.g. red) appears in a square subunit, the subunit is labeled as 1; otherwise labeled as 0. A square image of a red flower-like pattern with 750×750 pixels was sent to AI for the demonstration of our authentication system. R is determined by the lateral pixels of the image and will be even larger than 150. Based on the flower-like pattern shown in Figure 3a, the pattern filling density (D) in 30×30 arrays is in the range of 0.1-0.5. We use D value of 9/25 as an example to estimate the encoding capacity.

According to the binary-bit model established by Carro-Temboury *et al.*[3], the encoding capacity of a flower-like pattern ($\#codes_F$) can be expressed as follows:

$$\#codes_F = \left[C\left(1+L\left(\frac{1}{\sqrt{D}}-1\right)\right)^2+1\,\frac{R^2}{L^2}\right]^D \quad (1)$$

where C is the number of colors of a flower-liker pattern.

Based on eqn 1, the encoding capacity of a red flower-like pattern as shown in Supplementary Figure 16 is estimated to be $4.7\times10^{202} >> 10^{20}$. For a security label composed of 1,000 red flower-like patterns, its encoding capacity is $(4.7\times10^{202})^{1000} > 10^{202,000}$.

Supplementary Notes 2. Calculation of overall cost of a security label.

Material costs:

Cost of 1g PMMA: US$ 1.

Considering the concentration of 4 mg ml^{-1} for PMMA solution is enough for surface modification, and 1 ml PMMA solution can modify about 100 substrates (2 cm ×2 cm), the cost on PMMA is about US$ 4×10^{-5} for the fabrication of one security label. This cost can be completely ignored.

Cost of 1 ml of 20 mg ml^{-1} core-shell quantum dot inks: US$: 22

Since the volume of a single droplet is about 90 pl, and imaging one security label containing 1000 points, 1 ml core-shell quantum dots inks can produce 1.1×10^{4} security labels.

Instrument costs:

Plasma cleaning machine: US$ 8000 (PDC-MG Mingheng company)

Spin coater: US$ 500 (SC-1B Chuangshiweina company)

Ultrasonicator: US$ 30 (J JP-010T Jiemeng cleaning equipment co. LTD)

These machines can last for ten years even longer. If we assume that only a 1,000,000 security labels are produced by these machines, the instrument cost for each security label is less than US$ 9×10^{-3}.

To conclude, the overall cost of one unclonable inkjet-printed security label according to our strategy is about one cent (US$ 11×10^{-3}).

Supplementary References

1. Yang Y*, et al.* High-efficiency light-emitting devices based on quantum dots with tailored nanostructures. *Nat. Photonics* **9**, 259-266 (2015).

2. Horstmeyer R, Judkewitz B, Vellekoop IM, Assawaworrarit S, Yang C. Physical key-protected one-time pad. *Sci. Rep.* **3**, 3543 (2013).

3. Carro-Temboury MR, Arppe R, Vosch T, Sorensen TJ. An optical authentication system based on imaging of excitation-selected lanthanide luminescence. *Sci. Adv.* **4**, e1701384 (2018).

4. Wigger B, Meissner T, Forste A, Jetter V, Zimmermann A. Using unique surface patterns of injection moulded plastic components as an image based Physical Unclonable Function for secure component identification. *Sci. Rep.* **8**, 4738 (2018).

5. Bae HJ*, et al.* Biomimetic Microfingerprints for Anti-Counterfeiting Strategies. *Adv. Mater.* **27**, 2083-2089 (2015).

6. Smith AF, Patton P, Skrabalak SE. Plasmonic Nanoparticles as a Physically Unclonable Function for Responsive Anti-Counterfeit Nanofingerprints. *Adv. Funct. Mater.* **26**, 1315-1321 (2016).

7. Hu Z*, et al.* Physically unclonable cryptographic primitives using self-assembled carbon nanotubes. *Nat. Nanotechnol.* **11**, 559-565 (2016).

8. Nam H, Song K, Ha D, Kim T. Inkjet Printing Based Mono-layered Photonic Crystal Patterning for Anti-counterfeiting Structural Colors. *Sci. Rep.* **6**, 30885 (2016).

9. Tian L*, et al.* Plasmonic Nanogels for Unclonable Optical Tagging. *Acs Appl Mater Inter* **8**, 4031-4041 (2016).

10. Arppe-Tabbara R, Tabbara M, Sorensen TJ. Versatile and Validated Optical Authentication System Based on Physical Unclonable Functions. *Acs Appl Mater Inter* **11**, 6475-6482 (2019).
