# Supplementary material for: Inkjet-printed unclonable quantum dot fluorescent anti-counterfeiting labels with artificial intelligence authentication
Source: Nat Commun. 2019 Jun 3;10:2409. doi: 10.1038/s41467-019-10406-7 (PMC6547729; doi:10.1038/s41467-019-10406-7)
Supplement: Supplementary file 3 — Description of Additional Supplementary Files [file 41467_2019_10406_MOESM3_ESM.pdf]

Description of Additional Supplementary Files

File Name: Supplementary Data 1
Description: The original absorption and PL spectra of coumarin.

File Name: Supplementary Data 2
Description: Quantum dots for calculating PL quantum yield of the blue quantum dots.
