# Supplementary material for: Inkjet-printed unclonable quantum dot fluorescent anti-counterfeiting labels with artificial intelligence authentication
Source: Nat Commun. 2019 Jun 3;10:2409. doi: 10.1038/s41467-019-10406-7 (PMC6547729; doi:10.1038/s41467-019-10406-7)
Supplement: Supplementary file 4 — Supplementary Data 1 [file 41467_2019_10406_MOESM4_ESM.docx]

**Supplementary Data 1. Original absorption and PL spectra of Coumarin for calculating PL quantum yield of the blue quantum dots**

| **Coumarin Absorption** | |  | **Coumarin PL** | | |
| --- | --- | --- | --- | --- | --- |
| Wavelength (nm) | Absorption |  | Wavelength (nm) | PL | Integration |
| 345 | 0.0284 |  | 370 | 534.8669486 | 0 |
| 345.5 | 0.029 |  | 371 | 588.9953568 | 561.9311527 |
| 346 | 0.0297 |  | 372 | 759.882195 | 1236.369929 |
| 346.5 | 0.0305 |  | 373 | 705.2209172 | 1968.921485 |
| 347 | 0.0313 |  | 374 | 501.3686497 | 2572.216268 |
| 347.5 | 0.0321 |  | 375 | 804.5806961 | 3225.190941 |
| 348 | 0.0329 |  | 376 | 741.285333 | 3998.123956 |
| 348.5 | 0.0337 |  | 377 | 757.6146315 | 4747.573938 |
| 349 | 0.0341 |  | 378 | 851.5932278 | 5552.177868 |
| 349.5 | 0.035 |  | 379 | 1164.92752 | 6560.438242 |
| 350 | 0.0358 |  | 380 | 694.7611852 | 7490.282594 |
| 350.5 | 0.0367 |  | 381 | 1032.690294 | 8354.008334 |
| 351 | 0.0373 |  | 382 | 801.5759554 | 9271.141459 |
| 351.5 | 0.0383 |  | 383 | 1181.754129 | 10262.8065 |
| 352 | 0.039 |  | 384 | 1114.411865 | 11410.8895 |
| 352.5 | 0.0397 |  | 385 | 1145.072544 | 12540.6317 |
| 353 | 0.0404 |  | 386 | 1403.563684 | 13814.94982 |
| 353.5 | 0.0413 |  | 387 | 1152.242307 | 15092.85281 |
| 354 | 0.0422 |  | 388 | 1701.806366 | 16519.87715 |
| 354.5 | 0.0431 |  | 389 | 1537.15447 | 18139.35757 |
| 355 | 0.0438 |  | 390 | 1868.777515 | 19842.32356 |
| 355.5 | 0.0446 |  | 391 | 2036.35458 | 21794.88961 |
| 356 | 0.0455 |  | 392 | 2138.754953 | 23882.44437 |
| 356.5 | 0.0462 |  | 393 | 1962.434491 | 25933.0391 |
| 357 | 0.047 |  | 394 | 1722.887156 | 27775.69992 |
| 357.5 | 0.0478 |  | 395 | 2059.513912 | 29666.90045 |
| 358 | 0.0487 |  | 396 | 2242.452128 | 31817.88347 |
| 358.5 | 0.0497 |  | 397 | 2128.045936 | 34003.13251 |
| 359 | 0.0505 |  | 398 | 2330.214118 | 36232.26253 |
| 359.5 | 0.0512 |  | 399 | 2583.145621 | 38688.9424 |
| 360 | 0.0521 |  | 400 | 2583.396668 | 41272.21355 |
| 360.5 | 0.0529 |  | 401 | 2563.03647 | 43845.43012 |
| 361 | 0.0538 |  | 402 | 2908.293736 | 46581.09522 |
| 361.5 | 0.0546 |  | 403 | 3023.822924 | 49547.15355 |
| 362 | 0.0557 |  | 404 | 3296.160043 | 52707.14503 |
| 362.5 | 0.0566 |  | 405 | 3213.210812 | 55961.83046 |
| 363 | 0.0575 |  | 406 | 3575.808536 | 59356.34014 |
| 363.5 | 0.0584 |  | 407 | 3729.063973 | 63008.77639 |
| 364 | 0.0591 |  | 408 | 4132.219248 | 66939.418 |
| 364.5 | 0.06 |  | 409 | 4680.441732 | 71345.74849 |
| 365 | 0.0609 |  | 410 | 5134.008968 | 76252.97384 |
| 365.5 | 0.0616 |  | 411 | 4997.222599 | 81318.58962 |
| 366 | 0.0628 |  | 412 | 4933.311694 | 86283.85677 |
| 366.5 | 0.0638 |  | 413 | 5555.567947 | 91528.29659 |
| 367 | 0.0642 |  | 414 | 5772.128464 | 97192.1448 |
| 367.5 | 0.0652 |  | 415 | 6778.164799 | 103467.2914 |
| 368 | 0.066 |  | 416 | 6261.862802 | 109987.3052 |
| 368.5 | 0.0667 |  | 417 | 6430.866972 | 116333.6701 |
| 369 | 0.0676 |  | 418 | 7448.470248 | 123273.3387 |
| 369.5 | 0.0684 |  | 419 | 8008.668852 | 131001.9083 |
| 370 | 0.0693 |  | 420 | 8092.380924 | 139052.4332 |
| 370.5 | 0.0701 |  | 421 | 8675.831763 | 147436.5395 |
| 371 | 0.071 |  | 422 | 9699.700775 | 156624.3058 |
| 371.5 | 0.0717 |  | 423 | 10493.77951 | 166721.0459 |
| 372 | 0.0725 |  | 424 | 10854.19216 | 177395.0318 |
| 372.5 | 0.0733 |  | 425 | 12298.31073 | 188971.2832 |
| 373 | 0.0739 |  | 426 | 14134.83562 | 202187.8564 |
| 373.5 | 0.0746 |  | 427 | 14413.74251 | 216462.1454 |
| 374 | 0.0755 |  | 428 | 15655.9117 | 231496.9725 |
| 374.5 | 0.0759 |  | 429 | 16779.95142 | 247714.9041 |
| 375 | 0.0767 |  | 430 | 17847.36127 | 265028.5604 |
| 375.5 | 0.0772 |  | 431 | 20090.62162 | 283997.5519 |
| 376 | 0.078 |  | 432 | 22206.19823 | 305145.9618 |
| 376.5 | 0.0786 |  | 433 | 22997.67766 | 327747.8998 |
| 377 | 0.0793 |  | 434 | 25062.18447 | 351777.8308 |
| 377.5 | 0.0797 |  | 435 | 27491.419 | 378054.6326 |
| 378 | 0.0804 |  | 436 | 28758.66833 | 406179.6762 |
| 378.5 | 0.0806 |  | 437 | 31263.48537 | 436190.7531 |
| 379 | 0.0811 |  | 438 | 33882.98066 | 468763.9861 |
| 379.5 | 0.0816 |  | 439 | 36476.56821 | 503943.7605 |
| 380 | 0.082 |  | 440 | 37422.95512 | 540893.5222 |
| 380.5 | 0.0825 |  | 441 | 38666.40805 | 578938.2038 |
| 381 | 0.0828 |  | 442 | 42520.84927 | 619531.8324 |
| 381.5 | 0.0831 |  | 443 | 44138.82711 | 662861.6706 |
| 382 | 0.0835 |  | 444 | 45924.32406 | 707893.2462 |
| 382.5 | 0.0838 |  | 445 | 49089.72976 | 755400.2731 |
| 383 | 0.0841 |  | 446 | 51965.51363 | 805927.8948 |
| 383.5 | 0.0844 |  | 447 | 53214.90827 | 858518.1058 |
| 384 | 0.0845 |  | 448 | 56140.74738 | 913195.9336 |
| 384.5 | 0.0847 |  | 449 | 56364.97362 | 969448.7941 |
| 385 | 0.0848 |  | 450 | 59391.44908 | 1027327.005 |
| 385.5 | 0.0849 |  | 451 | 60401.48642 | 1087223.473 |
| 386 | 0.0852 |  | 452 | 63608.70984 | 1149228.571 |
| 386.5 | 0.0854 |  | 453 | 64689.58245 | 1213377.717 |
| 387 | 0.0855 |  | 454 | 66941.58781 | 1279193.303 |
| 387.5 | 0.0854 |  | 455 | 67653.31945 | 1346490.756 |
| 388 | 0.0856 |  | 456 | 68648.33078 | 1414641.581 |
| 388.5 | 0.0856 |  | 457 | 72240.29395 | 1485085.894 |
| 389 | 0.0855 |  | 458 | 73438.67187 | 1557925.377 |
| 389.5 | 0.0855 |  | 459 | 73139.21065 | 1631214.318 |
| 390 | 0.0853 |  | 460 | 76101.80401 | 1705834.825 |
| 390.5 | 0.0853 |  | 461 | 76797.61966 | 1782284.537 |
| 391 | 0.0853 |  | 462 | 75916.60782 | 1858641.651 |
| 391.5 | 0.085 |  | 463 | 75089.09887 | 1934144.504 |
| 392 | 0.0848 |  | 464 | 77650.71563 | 2010514.411 |
| 392.5 | 0.0846 |  | 465 | 78375.28449 | 2088527.411 |
| 393 | 0.0843 |  | 466 | 76707.83799 | 2166068.973 |
| 393.5 | 0.084 |  | 467 | 77044.94667 | 2242945.365 |
| 394 | 0.0836 |  | 468 | 76115.92387 | 2319525.8 |
| 394.5 | 0.0832 |  | 469 | 78109.57658 | 2396638.55 |
| 395 | 0.0828 |  | 470 | 77202.48328 | 2474294.58 |
| 395.5 | 0.0823 |  | 471 | 76555.34859 | 2551173.496 |
| 396 | 0.0817 |  | 472 | 77530.75376 | 2628216.548 |
| 396.5 | 0.0812 |  | 473 | 76503.2005 | 2705233.525 |
| 397 | 0.0806 |  | 474 | 75194.48439 | 2781082.367 |
| 397.5 | 0.0799 |  | 475 | 75783.96568 | 2856571.592 |
| 398 | 0.0795 |  | 476 | 73604.01922 | 2931265.585 |
| 398.5 | 0.0787 |  | 477 | 72851.55473 | 3004493.372 |
| 399 | 0.0779 |  | 478 | 72892.48364 | 3077365.391 |
| 399.5 | 0.077 |  | 479 | 70958.4235 | 3149290.844 |
| 400 | 0.0761 |  | 480 | 68736.8936 | 3219138.503 |
| 400.5 | 0.0751 |  | 481 | 68302.22467 | 3287658.062 |
| 401 | 0.0742 |  | 482 | 67408.74952 | 3355513.549 |
| 401.5 | 0.0731 |  | 483 | 66297.82049 | 3422366.834 |
| 402 | 0.072 |  | 484 | 65753.54593 | 3488392.517 |
| 402.5 | 0.0708 |  | 485 | 64569.08348 | 3553553.832 |
| 403 | 0.0697 |  | 486 | 62225.69419 | 3616951.221 |
| 403.5 | 0.0685 |  | 487 | 60910.84181 | 3678519.489 |
| 404 | 0.0673 |  | 488 | 60264.60517 | 3739107.212 |
| 404.5 | 0.066 |  | 489 | 56548.464 | 3797513.747 |
| 405 | 0.0647 |  | 490 | 56732.90593 | 3854154.432 |
| 405.5 | 0.0633 |  | 491 | 54898.2131 | 3909969.991 |
| 406 | 0.0618 |  | 492 | 53469.26602 | 3964153.731 |
| 406.5 | 0.0603 |  | 493 | 53666.16553 | 4017721.447 |
| 407 | 0.0591 |  | 494 | 51556.14227 | 4070332.601 |
| 407.5 | 0.0575 |  | 495 | 51296.84493 | 4121759.094 |
| 408 | 0.0562 |  | 496 | 50602.56279 | 4172708.798 |
| 408.5 | 0.0546 |  | 497 | 48155.33182 | 4222087.745 |
| 409 | 0.0532 |  | 498 | 46595.3666 | 4269463.095 |
| 409.5 | 0.0516 |  | 499 | 45883.62079 | 4315702.588 |
| 410 | 0.05 |  | 500 | 43806.88904 | 4360547.843 |
| 410.5 | 0.0484 |  | 501 | 43391.31236 | 4404146.944 |
| 411 | 0.047 |  | 502 | 42942.98182 | 4447314.091 |
| 411.5 | 0.0454 |  | 503 | 40689.82987 | 4489130.497 |
| 412 | 0.0441 |  | 504 | 40126.64505 | 4529538.734 |
| 412.5 | 0.0425 |  | 505 | 38817.2958 | 4569010.705 |
| 413 | 0.0414 |  | 506 | 37103.59258 | 4606971.149 |
| 413.5 | 0.0398 |  | 507 | 35674.38069 | 4643360.136 |
| 414 | 0.0385 |  | 508 | 35415.79583 | 4678905.224 |
| 414.5 | 0.0369 |  | 509 | 34539.07019 | 4713882.657 |
| 415 | 0.0357 |  | 510 | 32993.67267 | 4747649.028 |
| 415.5 | 0.0343 |  | 511 | 31709.03163 | 4780000.38 |
| 416 | 0.033 |  | 512 | 31918.66639 | 4811814.229 |
| 416.5 | 0.0316 |  | 513 | 30512.9624 | 4843030.044 |
| 417 | 0.0304 |  | 514 | 30565.27968 | 4873569.165 |
| 417.5 | 0.029 |  | 515 | 29413.16463 | 4903558.387 |
| 418 | 0.028 |  | 516 | 27061.31834 | 4931795.628 |
| 418.5 | 0.0267 |  | 517 | 26153.84836 | 4958403.212 |
| 419 | 0.0257 |  | 518 | 25451.97192 | 4984206.122 |
| 419.5 | 0.0246 |  | 519 | 26419.1814 | 5010141.699 |
| 420 | 0.0235 |  | 520 | 24767.17719 | 5035734.878 |
| 420.5 | 0.0224 |  | 521 | 24172.81438 | 5060204.874 |
| 421 | 0.0213 |  | 522 | 23482.06332 | 5084032.313 |
| 421.5 | 0.0204 |  | 523 | 23264.91736 | 5107405.803 |
| 422 | 0.0194 |  | 524 | 21098.55564 | 5129587.539 |
| 422.5 | 0.0185 |  | 525 | 22480.59559 | 5151377.115 |
| 423 | 0.0176 |  | 526 | 21097.80858 | 5173166.317 |
| 423.5 | 0.0167 |  | 527 | 18956.72015 | 5193193.581 |
| 424 | 0.0158 |  | 528 | 19375.84777 | 5212359.865 |
| 424.5 | 0.015 |  | 529 | 17216.67948 | 5230656.129 |
| 425 | 0.0143 |  | 530 | 16963.97111 | 5247746.454 |
| 425.5 | 0.0136 |  | 531 | 16333.95995 | 5264395.42 |
| 426 | 0.0129 |  | 532 | 16882.30355 | 5281003.552 |
| 426.5 | 0.0123 |  | 533 | 15581.44343 | 5297235.425 |
| 427 | 0.0115 |  | 534 | 15326.99418 | 5312689.644 |
| 427.5 | 0.011 |  | 535 | 14105.28813 | 5327405.785 |
| 428 | 0.0103 |  | 536 | 14396.71989 | 5341656.789 |
| 428.5 | 0.0098 |  | 537 | 14092.16431 | 5355901.231 |
| 429 | 0.0091 |  | 538 | 13797.54103 | 5369846.084 |
| 429.5 | 0.0087 |  | 539 | 12378.30185 | 5382934.005 |
| 430 | 0.0083 |  | 540 | 12643.81472 | 5395445.064 |
| 430.5 | 0.0078 |  | 541 | 12683.88479 | 5408108.913 |
| 431 | 0.0073 |  | 542 | 11691.83084 | 5420296.771 |
| 431.5 | 0.0069 |  | 543 | 12100.83107 | 5432193.102 |
| 432 | 0.0064 |  | 544 | 11500.27883 | 5443993.657 |
| 432.5 | 0.006 |  | 545 | 10385.48642 | 5454936.54 |
| 433 | 0.0056 |  | 546 | 10479.44829 | 5465369.007 |
| 433.5 | 0.0054 |  | 547 | 9773.913317 | 5475495.688 |
| 434 | 0.0051 |  | 548 | 9468.298052 | 5485116.794 |
| 434.5 | 0.0046 |  | 549 | 8846.442998 | 5494274.164 |
| 435 | 0.0044 |  | 550 | 8718.919045 | 5503056.845 |
| 435.5 | 0.0042 |  | 551 | 8518.290271 | 5511675.45 |
| 436 | 0.0038 |  | 552 | 8684.319021 | 5520276.754 |
| 436.5 | 0.0036 |  | 553 | 8230.215679 | 5528734.022 |
| 437 | 0.0033 |  | 554 | 7393.362915 | 5536545.811 |
| 437.5 | 0.003 |  | 555 | 7894.736839 | 5544189.861 |
| 438 | 0.0029 |  | 556 | 7779.992642 | 5552027.226 |
| 438.5 | 0.0028 |  | 557 | 6563.412598 | 5559198.928 |
| 439 | 0.0027 |  | 558 | 6950.454089 | 5565955.862 |
| 439.5 | 0.0025 |  | 559 | 6579.643171 | 5572720.91 |
| 440 | 0.0023 |  | 560 | 5770.092139 | 5578895.778 |
| 440.5 | 0.0022 |  | 561 | 5748.429533 | 5584655.039 |
| 441 | 0.002 |  | 562 | 5648.867265 | 5590353.687 |
| 441.5 | 0.0018 |  | 563 | 5893.867061 | 5596125.054 |
| 442 | 0.0017 |  | 564 | 5869.739461 | 5602006.858 |
| 442.5 | 0.0017 |  | 565 | 4920.606943 | 5607402.031 |
| 443 | 0.0016 |  | 566 | 5358.613608 | 5612541.641 |
| 443.5 | 0.0015 |  | 567 | 5323.665629 | 5617882.781 |
| 444 | 0.0014 |  | 568 | 4985.135151 | 5623037.181 |
| 444.5 | 0.0014 |  | 569 | 4477.750756 | 5627768.624 |
| 445 | 0.0012 |  | 570 | 4493.93626 | 5632254.467 |
| 445.5 | 0.0013 |  | 571 | 4199.778808 | 5636601.325 |
| 446 | 0.0011 |  | 572 | 4233.34688 | 5640817.888 |
| 446.5 | 0.0011 |  | 573 | 4224.959053 | 5645047.041 |
| 447 | 0.0008 |  | 574 | 4173.625119 | 5649246.333 |
| 447.5 | 0.0009 |  | 575 | 4142.278925 | 5653404.285 |
| 448 | 0.0008 |  | 576 | 3728.564351 | 5657339.707 |
| 448.5 | 0.0007 |  | 577 | 3263.279436 | 5660835.628 |
| 449 | 0.0007 |  | 578 | 3467.692076 | 5664201.114 |
| 449.5 | 0.0007 |  | 579 | 3343.919444 | 5667606.92 |
| 450 | 0.0007 |  | 580 | 3529.35997 | 5671043.56 |
| 450.5 | 0.0007 |  | 581 | 3446.754527 | 5674531.617 |
| 451 | 0.0006 |  | 582 | 2795.851154 | 5677652.92 |
| 451.5 | 0.0006 |  | 583 | 2476.351199 | 5680289.021 |
| 452 | 0.0006 |  | 584 | 3072.511491 | 5683063.452 |
| 452.5 | 0.0004 |  | 585 | 2865.356859 | 5686032.386 |
| 453 | 0.0005 |  | 586 | 2515.543687 | 5688722.837 |
| 453.5 | 0.0004 |  | 587 | 2749.870518 | 5691355.544 |
| 454 | 0.0004 |  | 588 | 2225.389483 | 5693843.174 |
| 454.5 | 0.0004 |  | 589 | 2628.724743 | 5696270.231 |
| 455 | 0.0005 |  | 590 | 2238.915316 | 5698704.051 |
| 455.5 | 0.0004 |  | 591 | 1842.333596 | 5700744.675 |
| 456 | 0.0005 |  | 592 | 1783.760786 | 5702557.723 |
| 456.5 | 0.0003 |  | 593 | 2518.880164 | 5704709.043 |
| 457 | 0.0003 |  | 594 | 2163.842606 | 5707050.404 |
| 457.5 | 0.0003 |  | 595 | 1903.693315 | 5709084.172 |
| 458 | 0.0004 |  | 596 | 2122.90309 | 5711097.471 |
| 458.5 | 0.0003 |  | 597 | 1729.553088 | 5713023.699 |
| 459 | 0.0005 |  | 598 | 1614.13678 | 5714695.544 |
| 459.5 | 0.0003 |  | 599 | 1496.654956 | 5716250.94 |
| 460 | 0.0003 |  | 600 | 1271.984898 | 5717635.259 |
