# Supplementary material for: Inkjet-printed unclonable quantum dot fluorescent anti-counterfeiting labels with artificial intelligence authentication
Source: Nat Commun. 2019 Jun 3;10:2409. doi: 10.1038/s41467-019-10406-7 (PMC6547729; doi:10.1038/s41467-019-10406-7)
Supplement: Supplementary file 5 — Supplementary Data 2 [file 41467_2019_10406_MOESM5_ESM.docx]

**Supplementary Data 2. Original absorption and PL spectra of the blue quantum dots calculating PL quantum yield of the blue quantum dots**

| **Blue quantum dots  Absorption** | |  | **Blue quantum dots PL** | | |
| --- | --- | --- | --- | --- | --- |
| Wavelength (nm) | Absorption |  | Wavelength (nm) | PL | integration |
| 345 | 0.0562 |  | 370 | 154.1958771 | 0 |
| 345.5 | 0.0555 |  | 371 | 176.4601474 | 165.3280122 |
| 346 | 0.0548 |  | 372 | 280.8260286 | 393.9711002 |
| 346.5 | 0.0541 |  | 373 | 184.4783194 | 626.6232742 |
| 347 | 0.0535 |  | 374 | 136.3168218 | 787.0208448 |
| 347.5 | 0.053 |  | 375 | 9.142962456 | 859.750737 |
| 348 | 0.0521 |  | 376 | 54.40626297 | 891.5253497 |
| 348.5 | 0.0515 |  | 377 | 188.8416292 | 1013.149296 |
| 349 | 0.0506 |  | 378 | 64.64974766 | 1139.894984 |
| 349.5 | 0.0499 |  | 379 | 130.4188305 | 1237.429273 |
| 350 | 0.0495 |  | 380 | 63.55859423 | 1334.417986 |
| 350.5 | 0.0489 |  | 381 | 194.7233818 | 1463.558974 |
| 351 | 0.0482 |  | 382 | 172.5463228 | 1647.193826 |
| 351.5 | 0.0476 |  | 383 | 313.9715767 | 1890.452776 |
| 352 | 0.0469 |  | 384 | 280.779552 | 2187.82834 |
| 352.5 | 0.0462 |  | 385 | 258.5647681 | 2457.5005 |
| 353 | 0.0456 |  | 386 | 193.0713568 | 2683.318563 |
| 353.5 | 0.045 |  | 387 | 225.2503757 | 2892.479429 |
| 354 | 0.0443 |  | 388 | 289.7611856 | 3149.985209 |
| 354.5 | 0.0438 |  | 389 | 397.2421665 | 3493.486886 |
| 355 | 0.0433 |  | 390 | 342.7169838 | 3863.466461 |
| 355.5 | 0.043 |  | 391 | 503.1752606 | 4286.412583 |
| 356 | 0.0425 |  | 392 | 759.3974459 | 4917.698936 |
| 356.5 | 0.0418 |  | 393 | 725.4801445 | 5660.137731 |
| 357 | 0.0412 |  | 394 | 606.3196434 | 6326.037625 |
| 357.5 | 0.0408 |  | 395 | 498.3725495 | 6878.383722 |
| 358 | 0.0405 |  | 396 | 464.6140171 | 7359.877005 |
| 358.5 | 0.04 |  | 397 | 579.031367 | 7881.699697 |
| 359 | 0.0394 |  | 398 | 387.3165291 | 8364.873645 |
| 359.5 | 0.0389 |  | 399 | 564.0147501 | 8840.539285 |
| 360 | 0.0384 |  | 400 | 634.8848724 | 9439.989096 |
| 360.5 | 0.0379 |  | 401 | 645.4590621 | 10080.16106 |
| 361 | 0.0377 |  | 402 | 802.2879272 | 10804.03456 |
| 361.5 | 0.0371 |  | 403 | 750.2090045 | 11580.28302 |
| 362 | 0.0366 |  | 404 | 969.8276855 | 12440.30137 |
| 362.5 | 0.0362 |  | 405 | 980.478771 | 13415.4546 |
| 363 | 0.0357 |  | 406 | 1084.021396 | 14447.70468 |
| 363.5 | 0.0354 |  | 407 | 1051.733767 | 15515.58226 |
| 364 | 0.0348 |  | 408 | 1624.116445 | 16853.50737 |
| 364.5 | 0.0343 |  | 409 | 1820.518947 | 18575.82506 |
| 365 | 0.0338 |  | 410 | 1860.479942 | 20416.32451 |
| 365.5 | 0.0334 |  | 411 | 2047.155303 | 22370.14213 |
| 366 | 0.0333 |  | 412 | 2202.51985 | 24494.97971 |
| 366.5 | 0.0328 |  | 413 | 2711.250225 | 26951.86475 |
| 367 | 0.0325 |  | 414 | 2866.318024 | 29740.64887 |
| 367.5 | 0.0322 |  | 415 | 2958.953609 | 32653.28469 |
| 368 | 0.0314 |  | 416 | 3453.675739 | 35859.59936 |
| 368.5 | 0.0313 |  | 417 | 4185.660998 | 39679.26773 |
| 369 | 0.0308 |  | 418 | 4460.385019 | 44002.29074 |
| 369.5 | 0.0304 |  | 419 | 4941.299041 | 48703.13277 |
| 370 | 0.0304 |  | 420 | 5379.770213 | 53863.6674 |
| 370.5 | 0.0299 |  | 421 | 6463.051283 | 59785.07814 |
| 371 | 0.0295 |  | 422 | 6893.951166 | 66463.57937 |
| 371.5 | 0.0291 |  | 423 | 8340.366369 | 74080.73814 |
| 372 | 0.0288 |  | 424 | 8920.04567 | 82710.94415 |
| 372.5 | 0.0285 |  | 425 | 9425.906872 | 91883.92043 |
| 373 | 0.0284 |  | 426 | 11106.5257 | 102150.1367 |
| 373.5 | 0.028 |  | 427 | 11332.21186 | 113369.5055 |
| 374 | 0.0275 |  | 428 | 12511.3222 | 125291.2725 |
| 374.5 | 0.0274 |  | 429 | 14960.53626 | 139027.2017 |
| 375 | 0.0273 |  | 430 | 15588.15045 | 154301.5451 |
| 375.5 | 0.0268 |  | 431 | 18228.24905 | 171209.7449 |
| 376 | 0.0266 |  | 432 | 19651.53852 | 190149.6386 |
| 376.5 | 0.0263 |  | 433 | 22649.65472 | 211300.2353 |
| 377 | 0.026 |  | 434 | 24172.34334 | 234711.2343 |
| 377.5 | 0.0258 |  | 435 | 27916.61641 | 260755.7142 |
| 378 | 0.0253 |  | 436 | 32034.59118 | 290731.318 |
| 378.5 | 0.0249 |  | 437 | 33828.93138 | 323663.0792 |
| 379 | 0.0249 |  | 438 | 37536.27854 | 359345.6842 |
| 379.5 | 0.0246 |  | 439 | 42497.3048 | 399362.4759 |
| 380 | 0.0244 |  | 440 | 45754.60754 | 443488.432 |
| 380.5 | 0.024 |  | 441 | 50571.96752 | 491651.7196 |
| 381 | 0.0237 |  | 442 | 56526.27462 | 545200.8406 |
| 381.5 | 0.0236 |  | 443 | 61545.7168 | 604236.8363 |
| 382 | 0.0231 |  | 444 | 66967.2037 | 668493.2966 |
| 382.5 | 0.0229 |  | 445 | 74403.40713 | 739178.602 |
| 383 | 0.0226 |  | 446 | 84003.60365 | 818382.1074 |
| 383.5 | 0.0227 |  | 447 | 92724.43166 | 906746.1251 |
| 384 | 0.0224 |  | 448 | 103136.9112 | 1004676.796 |
| 384.5 | 0.0219 |  | 449 | 110513.4481 | 1111501.976 |
| 385 | 0.0217 |  | 450 | 122104.3667 | 1227810.884 |
| 385.5 | 0.0217 |  | 451 | 132487.963 | 1355107.048 |
| 386 | 0.0214 |  | 452 | 146525.9861 | 1494614.023 |
| 386.5 | 0.0212 |  | 453 | 159880.5543 | 1647817.293 |
| 387 | 0.0209 |  | 454 | 172015.9983 | 1813765.569 |
| 387.5 | 0.0209 |  | 455 | 184320.322 | 1991933.73 |
| 388 | 0.0206 |  | 456 | 196341.9543 | 2182264.868 |
| 388.5 | 0.0204 |  | 457 | 204218.3075 | 2382544.999 |
| 389 | 0.0204 |  | 458 | 212364.9132 | 2590836.609 |
| 389.5 | 0.0201 |  | 459 | 214509.4064 | 2804273.769 |
| 390 | 0.02 |  | 460 | 213938.2639 | 3018497.604 |
| 390.5 | 0.0197 |  | 461 | 212808.3083 | 3231870.89 |
| 391 | 0.0196 |  | 462 | 204035.495 | 3440292.792 |
| 391.5 | 0.0195 |  | 463 | 189227.8291 | 3636924.454 |
| 392 | 0.019 |  | 464 | 171880.03 | 3817478.383 |
| 392.5 | 0.0187 |  | 465 | 154990.9802 | 3980913.888 |
| 393 | 0.0188 |  | 466 | 135391.7344 | 4126105.246 |
| 393.5 | 0.0185 |  | 467 | 118557.5228 | 4253079.874 |
| 394 | 0.0183 |  | 468 | 100809.1872 | 4362763.229 |
| 394.5 | 0.0181 |  | 469 | 82505.64539 | 4454420.645 |
| 395 | 0.0178 |  | 470 | 70533.38755 | 4530940.162 |
| 395.5 | 0.0176 |  | 471 | 58406.67892 | 4595410.195 |
| 396 | 0.0177 |  | 472 | 47936.44369 | 4648581.756 |
| 396.5 | 0.0176 |  | 473 | 39887.82941 | 4692493.893 |
| 397 | 0.0173 |  | 474 | 34158.66881 | 4729517.142 |
| 397.5 | 0.0171 |  | 475 | 27642.40072 | 4760417.677 |
| 398 | 0.0172 |  | 476 | 22381.24502 | 4785429.5 |
| 398.5 | 0.017 |  | 477 | 19320.73419 | 4806280.489 |
| 399 | 0.0166 |  | 478 | 15243.43084 | 4823562.572 |
| 399.5 | 0.0166 |  | 479 | 12465.35728 | 4837416.966 |
| 400 | 0.0165 |  | 480 | 10819.6533 | 4849059.471 |
| 400.5 | 0.0162 |  | 481 | 8238.482207 | 4858588.539 |
| 401 | 0.016 |  | 482 | 7016.740835 | 4866216.151 |
| 401.5 | 0.0158 |  | 483 | 5880.76779 | 4872664.905 |
| 402 | 0.0158 |  | 484 | 4420.800818 | 4877815.689 |
| 402.5 | 0.0155 |  | 485 | 3575.40237 | 4881813.791 |
| 403 | 0.0156 |  | 486 | 3218.407662 | 4885210.696 |
| 403.5 | 0.0156 |  | 487 | 2256.482723 | 4887948.141 |
| 404 | 0.0153 |  | 488 | 1870.746881 | 4890011.756 |
| 404.5 | 0.015 |  | 489 | 1590.069032 | 4891742.164 |
| 405 | 0.0148 |  | 490 | 1450.963368 | 4893262.68 |
| 405.5 | 0.0148 |  | 491 | 1193.023619 | 4894584.673 |
| 406 | 0.0147 |  | 492 | 1173.171404 | 4895767.771 |
| 406.5 | 0.0147 |  | 493 | 792.0405732 | 4896750.377 |
| 407 | 0.0146 |  | 494 | 746.4552508 | 4897519.625 |
| 407.5 | 0.0144 |  | 495 | 821.7385139 | 4898303.722 |
| 408 | 0.0142 |  | 496 | 545.0292862 | 4898987.106 |
| 408.5 | 0.0139 |  | 497 | 400.5413815 | 4899459.891 |
| 409 | 0.0138 |  | 498 | 414.3008868 | 4899867.312 |
| 409.5 | 0.0136 |  | 499 | 268.21015 | 4900208.568 |
| 410 | 0.0136 |  | 500 | 281.5574985 | 4900483.451 |
| 410.5 | 0.0134 |  | 501 | 158.9880341 | 4900703.724 |
| 411 | 0.0133 |  | 502 | 197.3873023 | 4900881.912 |
| 411.5 | 0.0131 |  | 503 | 110.5701899 | 4901035.891 |
| 412 | 0.0132 |  | 504 | 161.7500336 | 4901172.051 |
| 412.5 | 0.0131 |  | 505 | 188.087997 | 4901346.97 |
| 413 | 0.0131 |  | 506 | 176.4767276 | 4901529.252 |
| 413.5 | 0.0128 |  | 507 | 177.5741067 | 4901706.277 |
| 414 | 0.0128 |  | 508 | 165.7242767 | 4901877.927 |
| 414.5 | 0.0126 |  | 509 | 166.7421356 | 4902044.16 |
| 415 | 0.0127 |  | 510 | 154.6537449 | 4902204.858 |
| 415.5 | 0.0123 |  | 511 | 102.8657656 | 4902333.618 |
| 416 | 0.0124 |  | 512 | 262.7378376 | 4902516.419 |
| 416.5 | 0.0122 |  | 513 | 144.193574 | 4902719.885 |
| 417 | 0.0121 |  | 514 | 239.1060817 | 4902911.535 |
| 417.5 | 0.0119 |  | 515 | 145.9576263 | 4903104.067 |
| 418 | 0.0119 |  | 516 | 187.8124085 | 4903270.952 |
| 418.5 | 0.0118 |  | 517 | 189.1234056 | 4903459.42 |
| 419 | 0.0117 |  | 518 | 80.03764753 | 4903594 |
| 419.5 | 0.0116 |  | 519 | 122.2724289 | 4903695.155 |
| 420 | 0.0116 |  | 520 | 53.16045713 | 4903782.872 |
| 420.5 | 0.0116 |  | 521 | 25.36496787 | 4903822.134 |
| 421 | 0.0113 |  | 522 | 210.066814 | 4903939.85 |
| 421.5 | 0.0112 |  | 523 | 140.0984332 | 4904114.933 |
| 422 | 0.0111 |  | 524 | 11.51825066 | 4904190.741 |
| 422.5 | 0.0111 |  | 525 | 156.5985761 | 4904274.8 |
| 423 | 0.0108 |  | 526 | 128.5382965 | 4904417.368 |
| 423.5 | 0.0107 |  | 527 | 41.19746695 | 4904502.236 |
| 424 | 0.0106 |  | 528 | 293.4387225 | 4904669.554 |
| 424.5 | 0.0106 |  | 529 | 116.4297728 | 4904874.488 |
| 425 | 0.0104 |  | 530 | 162.3634243 | 4905013.885 |
| 425.5 | 0.0104 |  | 531 | 87.76817465 | 4905138.951 |
| 426 | 0.0102 |  | 532 | 179.7286015 | 4905272.699 |
| 426.5 | 0.0101 |  | 533 | 242.2144885 | 4905483.671 |
| 427 | 0.0101 |  | 534 | 135.7736549 | 4905672.665 |
| 427.5 | 0.0098 |  | 535 | 183.2258916 | 4905832.165 |
| 428 | 0.0098 |  | 536 | 90.72248521 | 4905969.139 |
| 428.5 | 0.0097 |  | 537 | 217.4327677 | 4906123.216 |
| 429 | 0.0095 |  | 538 | 203.1384003 | 4906333.502 |
| 429.5 | 0.0095 |  | 539 | 172.6312757 | 4906521.387 |
| 430 | 0.0095 |  | 540 | 125.5688245 | 4906670.487 |
| 430.5 | 0.0094 |  | 541 | 240.2373878 | 4906853.39 |
| 431 | 0.0092 |  | 542 | 242.2159804 | 4907094.617 |
| 431.5 | 0.0089 |  | 543 | 211.1953064 | 4907321.322 |
| 432 | 0.0088 |  | 544 | -36.5933084 | 4907408.623 |
| 432.5 | 0.0087 |  | 545 | 164.3167048 | 4907472.485 |
| 433 | 0.0087 |  | 546 | 216.2104095 | 4907662.748 |
| 433.5 | 0.0085 |  | 547 | 166.7554849 | 4907854.231 |
| 434 | 0.0084 |  | 548 | 116.5539948 | 4907995.886 |
| 434.5 | 0.0082 |  | 549 | 117.4001022 | 4908112.863 |
| 435 | 0.0082 |  | 550 | 292.1376944 | 4908317.632 |
| 435.5 | 0.0079 |  | 551 | 66.54914274 | 4908496.976 |
| 436 | 0.0078 |  | 552 | 296.2968309 | 4908678.399 |
| 436.5 | 0.0077 |  | 553 | 280.6158993 | 4908966.855 |
| 437 | 0.0077 |  | 554 | 121.6131297 | 4909167.969 |
| 437.5 | 0.0077 |  | 555 | 212.4951978 | 4909335.024 |
| 438 | 0.0074 |  | 556 | 159.8131075 | 4909521.178 |
| 438.5 | 0.0073 |  | 557 | 161.15522 | 4909681.662 |
| 439 | 0.0072 |  | 558 | 125.5661206 | 4909825.023 |
| 439.5 | 0.007 |  | 559 | 405.874989 | 4910090.743 |
| 440 | 0.007 |  | 560 | 146.4109261 | 4910366.886 |
| 440.5 | 0.0069 |  | 561 | 261.4644942 | 4910570.824 |
| 441 | 0.0068 |  | 562 | 53.54376554 | 4910728.328 |
| 441.5 | 0.0065 |  | 563 | 73.33560848 | 4910791.768 |
| 442 | 0.0064 |  | 564 | 268.7538307 | 4910962.812 |
| 442.5 | 0.0063 |  | 565 | 290.8345957 | 4911242.607 |
| 443 | 0.0062 |  | 566 | 174.3927505 | 4911475.22 |
| 443.5 | 0.006 |  | 567 | 255.7917419 | 4911690.312 |
| 444 | 0.0059 |  | 568 | 116.8705896 | 4911876.644 |
| 444.5 | 0.0058 |  | 569 | 280.3673341 | 4912075.263 |
| 445 | 0.0055 |  | 570 | 262.1804199 | 4912346.536 |
| 445.5 | 0.0054 |  | 571 | 222.9971048 | 4912589.125 |
| 446 | 0.0053 |  | 572 | 391.2827991 | 4912896.265 |
| 446.5 | 0.0052 |  | 573 | 310.4736544 | 4913247.143 |
| 447 | 0.0048 |  | 574 | 101.4863251 | 4913453.123 |
| 447.5 | 0.0049 |  | 575 | -68.18566132 | 4913469.774 |
| 448 | 0.0048 |  | 576 | 253.7315994 | 4913562.547 |
| 448.5 | 0.0044 |  | 577 | 190.9365627 | 4913784.881 |
| 449 | 0.0043 |  | 578 | 192.6495598 | 4913976.674 |
| 449.5 | 0.0041 |  | 579 | 128.1025943 | 4914137.05 |
| 450 | 0.0041 |  | 580 | 307.4821186 | 4914354.842 |
| 450.5 | 0.0039 |  | 581 | 62.9133812 | 4914540.04 |
| 451 | 0.0038 |  | 582 | 199.379985 | 4914671.187 |
| 451.5 | 0.0036 |  | 583 | 86.80935936 | 4914814.281 |
| 452 | 0.0036 |  | 584 | 110.5551361 | 4914912.964 |
| 452.5 | 0.0034 |  | 585 | 181.11656 | 4915058.799 |
| 453 | 0.0034 |  | 586 | 323.2262838 | 4915310.971 |
| 453.5 | 0.0033 |  | 587 | -4.724863434 | 4915470.222 |
| 454 | 0.0031 |  | 588 | 138.1933512 | 4915536.956 |
| 454.5 | 0.0028 |  | 589 | 139.3656628 | 4915675.735 |
| 455 | 0.0029 |  | 590 | 43.61523342 | 4915767.226 |
| 455.5 | 0.0028 |  | 591 | 263.8886318 | 4915920.978 |
| 456 | 0.0026 |  | 592 | 68.98522377 | 4916087.415 |
| 456.5 | 0.0024 |  | 593 | 144.0779581 | 4916193.946 |
| 457 | 0.0024 |  | 594 | 45.08005428 | 4916288.525 |
| 457.5 | 0.0021 |  | 595 | -5.049584389 | 4916308.54 |
| 458 | 0.002 |  | 596 | 122.1814728 | 4916367.106 |
| 458.5 | 0.0018 |  | 597 | -56.4542551 | 4916399.97 |
| 459 | 0.0019 |  | 598 | 305.2374039 | 4916524.362 |
| 459.5 | 0.0016 |  | 599 | 73.00755882 | 4916713.484 |
| 460 | 0.0014 |  | 600 | 99.86658287 | 4916799.921 |
